# Patients’ Adoption of Electronic Personal Health Records in England: Secondary Data Analysis

**DOI:** 10.2196/17499

**Published:** 2020-10-07

**Authors:** Alaa Abd-Alrazaq, Ali Abdallah Alalwan, Brian McMillan, Bridgette M Bewick, Mowafa Househ, Alaa T AL-Zyadat

**Affiliations:** 1 Division of Information and Computing Technology College of Science and Engineering Hamad Bin Khalifa University Doha Qatar; 2 Amman University College for Banking and Financial Sciences Al-Balqa Applied University Amman Jordan; 3 Centre for Primary Care and Health Services Research University of Manchester Manchester United Kingdom; 4 Leeds Institute of Health Sciences School of Medicine University of Leeds Leeds United Kingdom

**Keywords:** health records, personal, patient portal, medical informatics

## Abstract

**Background:**

In England, almost all general practices (GPs) have implemented GP online services such as electronic personal health records (ePHRs) that allow people to schedule appointments, request repeat prescriptions, and access parts of their medical records. The overall adoption rate of GP online services has been low, reaching just 28% in October 2019. In a previous study, Abd-Alrazaq et al adopted a model to assess the factors that influence patients’ use of GP online services in England. According to the previous literature, the predictive power of the Abd-Alrazaq model could be improved by proposing new associations between the existing variables in the model.

**Objective:**

This study aims to improve the predictive power of the Abd-Alrazaq model by proposing new relationships between the existing variables in the model.

**Methods:**

The Abd-Alrazaq model was amended by proposing new direct, mediating, moderating, and moderated mediating effects. The amended model was examined using data from a previous study, which were collected by a cross-sectional survey of a convenience sample of 4 GPs in West Yorkshire, England. Structural equation modeling was used to examine the theoretical model and hypotheses.

**Results:**

The new model accounted for 53% of the variance in performance expectancy (PE), 76% of the variance in behavioral intention (BI), and 49% of the variance in use behavior (UB). In addition to the significant associations found in the previous study, this study found that social influence (SI) and facilitating conditions (FCs) are associated with PE directly and BI indirectly through PE. The association between BI and UB was stronger for younger women with higher levels of education, income, and internet access. The indirect effects of effort expectancy (EE), perceived privacy and security (PPS), and SI on BI were statistically stronger for women without internet access, patients with internet access, and patients without internet access, respectively. The indirect effect of PPS on BI was stronger for patients with college education or diploma than for those with secondary school education and lower, whereas the indirect effect of EE on BI was stronger for patients with secondary school education or lower than for those with college education or a diploma.

**Conclusions:**

The predictive power of the Abd-Alrazaq model improved by virtue of new significant associations that were not examined before in the context of ePHRs. Further studies are required to validate the new model in different contexts and to improve its predictive power by proposing new variables. The influential factors found in this study should be considered to improve patients’ use of ePHRs.

## Introduction

### Background

An electronic personal health record (ePHR) has been defined by the Markle Foundation as “an electronic application through which individuals can access, manage and share their health information, and that of others for whom they are authorized, in a private, secure and confidential environment” [1]. Several services can also be provided by more advanced ePHRs, such as requesting repeat prescriptions, booking appointments, viewing test results, and messaging providers [2-4]. ePHRs have the potential to empower patients [5,6], improve patient self-management and medication adherence [7,8], enhance the rapport and communication between patients and health care providers [9,10], ease access to health services [11,12], avoid duplicated tests and medical images [9,11], and decrease adverse events [4,9,11,13].

In England, almost all general practices (GPs) have implemented GP online services, that is, ePHRs that allow people to schedule appointments, request repeat prescriptions, and access coded information in their medical records, such as demographics, medications, allergies, and test results [14]. The number of providers offering GP online services is growing [15].

### Research Problems and Aims

Despite the aforementioned potential benefits of ePHRs, the overall adoption rate of GP online services has been low, reaching just 28% in October 2019 [16]. To improve the adoption and implementation of ePHRs, it is important to identify the factors that influence individuals’ use of the system [17-23]. A recent systematic review of 97 studies found that more than 150 factors could affect patients’ acceptance and adoption of ePHRs [24]. Unfortunately, none of these studies were carried out in the United Kingdom and included a number of shortcomings, namely, few studies were theory based [21,25-28], many focused on factors affecting patients’ intention to use ePHRs instead of actual use [29-32], many assessed factors affecting self-reported use rather than actual use [28,33-36], almost all examined independent and dependent variables at one point in time using the same data collection instrument and were therefore at risk of common method bias [26,33,37], and almost all the studies did not differentiate between factors affecting initial use and continuing use of ePHRs. Therefore, Abd-Alrazaq et al [38] conducted a cross-sectional survey to assess the factors that influence patients’ use of ePHRs in England. The study identified several significant factors (performance expectancy [PE], effort expectancy [EE], perceived privacy and security [PPS], behavioral intention [BI], and some moderators), which were able to predict 48% of the variance in use behavior (UB). On the basis of previous research, we propose an amended model that we expect will predict UB more accurately. This study aims to improve the predictive power of the Abd-Alrazaq model by proposing new relationships between the variables existing in the model.

### Theoretical Foundation

In a previous study [38], the Unified Theory of Acceptance and Use of Technology (UTAUT) [39] was selected from 12 models as the theoretical foundation. The selection process was based on 6 predefined criteria: 4 criteria related to the goodness of the theory (ie, logical consistency, explanatory power, falsifiability, and parsimony), and 2 criteria related to the applicability of the theory on the phenomena of interest (ie, population and type of behavior). Multimedia Appendix 1 [27,29,37,39-65] elaborates on the selection process of the appropriate theory. Abd-Alrazaq et al [38] adapted UTAUT to the context of ePHRs by removing experience and voluntariness and adding PPS, education, income, and internet access to the model (Multimedia Appendix 2). Their justifications for these adaptations are provided in Multimedia Appendix 3.

Given that the study did not find a significant association between social influence (SI) and BI, the authors recommended that researchers examine other associations of SI [38]. Several studies have found that SI positively affects PE [66-68]. In other words, individuals who perceive that using technology is recommended by those important to them are more likely to perceive that the technology is useful. Therefore, this study proposes that SI directly affects PE and indirectly affects BI through PE.

The 2019 Abd-Alrazaq model [38] could be criticized for failing to hypothesize that facilitating conditions (FCs) are associated with BI. This argument is in line with the findings of Venkatesh et al [69], who proposed this relationship in the extended Unified Theory of Acceptance and Use of Technology (UTAUT2) framework, which is suitable for the consumer context [69]. Several studies have found that FCs are also associated with PE [70-72]. Accordingly, this study proposed that FCs directly affect both PE and BI and indirectly affect BI through PE.

**Figure 1:**
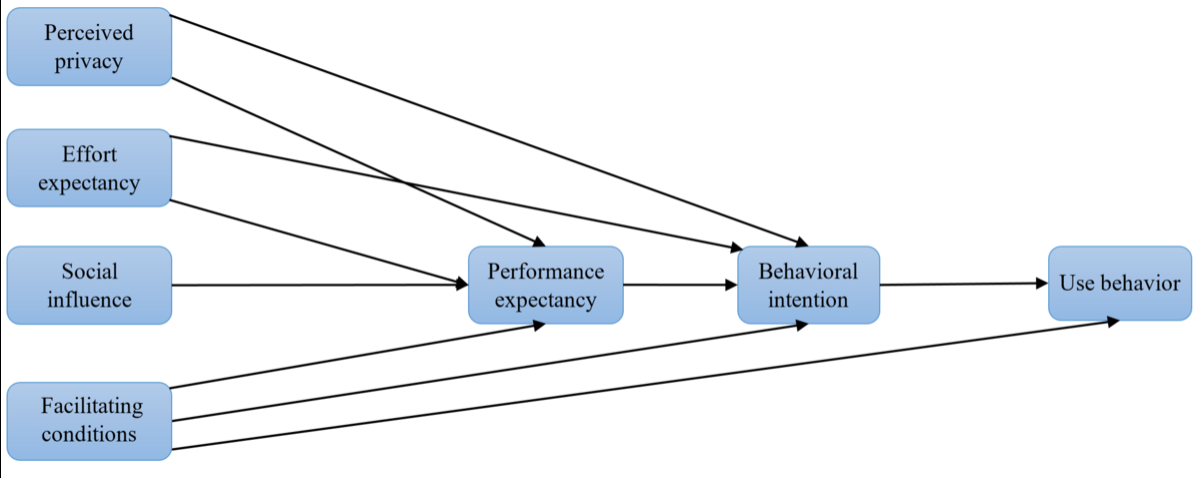
The proposed conceptual model.

The study by Abd-Alrazaq et al [38] also highlighted the need to assess the effect of moderators on indirect relationships (ie, moderated mediation) in the context of ePHRs. To address this recommendation, this study hypothesized that all mediating effects are moderated by sex, education, income, and internet access. To the best of our knowledge, no studies have assessed the moderating effects of these variables on the relationship between BI and UB. Thus, we explored the moderating effect of age, sex, education, income, and internet access on the relationship between BI and UB. Our proposed model and hypotheses are presented in Figure 1 and Table 1, respectively. The conceptual definitions of the constructs in the proposed model are presented in Multimedia Appendix 4.

**Table 1:** The proposed research hypotheses.

Hypothesis #	Hypothesis
H1	PPS^a^ positively affects PE^b^.
H2	PPS positively affects BI^c^.
H3	PPS indirectly and positively affects BI through PE.
H4	The positive relationship between PPS and PE is moderated by age, sex, education, income, and internet access, such that the influence is stronger for older women with a higher level of education and lower income and with internet access.
H5	The positive relationship between PPS and BI is moderated by age, sex, education, income, and internet access, such that the influence is stronger for older women with a higher level of education and lower income and with internet access.
H6	The indirect effect of PPS on BI is moderated by sex, education, income, and internet access, such that the influence is stronger for women with a higher level of education and lower income and with internet access.
H7	EE^d^ positively affects PE.
H8	EE positively affects BI.
H9	EE indirectly and positively affects BI through PE.
H10	The positive relationship between EE and PE is moderated by age, sex, education, income, and internet access, such that the influence is stronger for older women with a lower level of education and income and without internet access.
H11	The positive relationship between EE and BI is moderated by age, sex, education, income, and internet access, such that the influence is stronger for older women with a lower level of education and income and without internet access.
H12	The indirect effect of EE on BI is moderated by sex, education, income, and internet access, such that the influence is stronger for women with a lower level of education and income and without internet access.
H13	SI^e^ positively affects PE.
H14	SI indirectly and positively affects BI through PE.
H15	The positive relationship between SI and PE is moderated by age, sex, education, income, and internet access, such that the influence is stronger for older women with a lower level of education and income and with internet access.
H16	The indirect effect of SI on BI is moderated by age, sex, education, income, and internet access, such that the influence is stronger for older women with a lower level of education and income and with internet access.
H17	FCs^f^ positively affect PE.
H18	FCs positively affect BI.
H19	FCs positively affect UB^g^.
H20	FCs indirectly and positively affect BI through PE.
H21	The positive relationship between FCs and PE is moderated by age, sex, education, income, and internet access, such that the influence is stronger for older women with a lower level of education and income and without internet access.
H22	The positive relationship between FCs and BI is moderated by age, sex, education, income, and internet access, such that the influence is stronger for older women with a lower level of education and income and without internet access.
H23	The positive relationship between FCs and UB is moderated by age, sex, education, income, and internet access, such that the influence is stronger for older women with a lower level of education and income and without internet access.
H24	The indirect effect of FCs on BI is moderated by age, sex, education, income, and internet access, such that the influence is stronger for older women with a lower level of education and income and without internet access.
H25	The indirect effect of FCs on UB is moderated by age, sex, education, income, and internet access, such that the influence is stronger for older women with a lower level of education and income and without internet access.
H26	PE positively affects BI.
H27	The positive relationship between PE and BI is moderated by age and sex, such that the influence is stronger for younger men with a lower level of education, higher income, and internet access
H28	BI positively affects UB.
H29	The positive relationship between BI and UB is moderated by age, sex, education, income, and internet access, such that the influence is stronger for younger women with a higher level of education and income and with internet access.

^a^PPS: perceived privacy and security.

^b^PE: performance expectancy.

^c^BI: behavioral intention.

^d^EE: effort expectancy.

^e^SI: social influence.

^f^FC: facilitating condition.

^g^UB: use behavior.

## Methods

### Study Design and Setting

This study used secondary data analysis from data collected by Abd-Alrazaq et al [38] using a cross-sectional survey of 4 West Yorkshire GPs (Multimedia Appendix 5). Health Research Authority approval was granted before starting data collection (REC reference: 17/SC/0323).

### Measurement

All variables except UB were measured using self-administered questionnaires. The questionnaires were composed of 29 questions adopted from previous research (Multimedia Appendix 6). A panel of experts evaluated the face validity and content validity of the questions, and based on their suggestions, the questionnaire was amended and sent via email to 37 patients for pilot testing. The questionnaire was subsequently amended slightly because of the issues reported by patients (Multimedia Appendix 7). UB was measured objectively using system logs by extracting data on the number of times that each participant logged into the system during the 6 months after completing the questionnaire. One open-ended question was added to the questionnaire to obtain qualitative data that enabled the exploration of additional factors. The qualitative data were analyzed using thematic analysis, and the results have been reported elsewhere.

### Recruitment

A convenience sampling approach was used to recruit patients. Patients were eligible to participate if they were living in England, were registered at 1 of the 4 GP practices, were aged 18 years or older, and had not used GP online services before (nonusers). The questionnaire was delivered to eligible participants visiting 1 of the 4 GP practices during the study period. Data on participants’ use of GP online services were extracted from the system logs after 6 months of completing the questionnaire.

### Statistical Analysis

Before assessing the proposed model, it is a prerequisite to check normality [73,74], linearity [73], multicollinearity [73], and common method bias [75,76]. Univariate normality was examined by assessing skewness and kurtosis [73,77]. This study checked the linearity between each proposed relationship using scatterplot graphs [73] and the curve estimation procedure [78]. Multicollinearity was assessed in this study using tolerance, which refers to the proportion of the variability of one predictor that is unexplained by other predictors [73,77]. We checked the common method bias using Harman single-factor test [75]. All the aforementioned analyses were carried out using SPSS v.22 (IBM).

The theoretical model and hypotheses were examined using structural equation modeling (SEM). In SEM, models consist of 2 elements: a measurement model in which the relationships between observed variables and latent variables are examined, and a structural model in which the relationships proposed among the latent variables are assessed [73,79]. Although the measurement model in this study is identical to the original study [38], it was reassessed just for the sake of completeness. The measurement model was examined in terms of 3 aspects: model fit, construct reliability, and construct validity [73,77]. The structural model was then assessed for model fit, predictive power, and strength of relationships [77,79,80]. The strength of relationships was tested using different approaches depending on the type of the proposed effect. To be more precise, path coefficients were checked to examine direct effects [81]. Mediating effects were examined by assessing the indirect effects of using bootstrapping. The moderating effect for the metric moderator (ie, age) was examined using the interaction effect method [73,82]. The moderating effects for the nonmetric moderator (sex) were tested using multigroup SEM [73,74,82]. Moderated mediating effects were assessed using multigroup SEM for indirect effects. All analyses were conducted using the Analysis of Moment Structures v.24 (IBM) software.

## Results

### Participants’ Characteristics

The response rate was 78.0% (624/800). As shown in Multimedia Appendix 8, the mean age of participants was 44.2 (SD 1.89) years. Most participants were White (498/624, 79.8%) and had internet access (528/624, 84.6%). About half of the sample (284/624, 45.5%) had an income level of less than US $25,000 per year. The most prominent education levels among respondents were bachelor’s degrees (174/624, 27.9%), college or diploma (165/624, 26.4%), and secondary school (147/624, 23.6%). There were no significant differences between participants and the target population in terms of age, sex, and ethnicity (*P*=.21, *P*=.06, and *P*=.64, respectively; Multimedia Appendix 8). Thus, the risk of nonresponse bias is minimal.

### Normality, Linearity, Multicollinearity, and Common Method Bias

Histograms presented in Multimedia Appendix 9 show no severe skewness and kurtosis for all items. This finding was confirmed by the absolute values of skewness and kurtosis, which were considerably less than the cutoff points of 3 and 10, respectively [77] (Multimedia Appendix 10).

According to the scatterplots shown in Multimedia Appendix 11, there was an indication of possible nonlinearity for only 2 relationships: the effect of BI and FCs on UB. However, the results of the curve estimation procedure showed that the F values for all proposed relationships in the linear model were significant and higher than the F values of the proposed relationships in the 10 nonlinear models, indicating that all proposed relationships between variables are linear (Multimedia Appendix 12).

As shown in Multimedia Appendix 13, all values of tolerance are within the predetermined cutoff point (≥0.2) [83], indicating that there is no serious multicollinearity between independent variables.

With regard to the common method bias, 5 factors emerged from the Harman single-factor test; a single factor was able to explain less than half of the variance (47.3%; Multimedia Appendix 14). This means that there are no concerns regarding the presence of the common method bias in this study.

### Measurement Model

#### Model Fit

Nine indices were used to assess the absolute model fit (chi-square/df, goodness-of-fit index, adjusted goodness-of-fit index, root mean square error of approximation, p of Close Fit, and standardized root mean square residual) and incremental fit (normed-fit index, comparative fit index, and Tucker-Lewis index) [73,77]. Given that the measurement model in this study is identical to the modified measurement model in the original study, the results of the fit indices were the same between the 2 studies and were within their suggested levels (Multimedia Appendix 15). This indicates that the measurement model adequately fits the collected data.

#### Construct Reliability

Three measures were used to assess the construct reliability: Cronbach alpha (α), composite reliability (CR), and average variance extracted (AVE). Yielded values of α, CR, and AVE for each construct were within their recommended values of ≥.70, ≥.70, and ≥.50, respectively (Multimedia Appendix 16) [73,77]. This means that the measurement items are consistent and reproducible in measuring what it is assumed to measure.

#### Construct Validity

Two components of construct validity were examined in this study: convergent validity and discriminant validity [73,77]. The convergent validity was examined by checking factor loadings and the AVE [73]. As shown in Multimedia Appendix 17, the values of factor loading and AVE for all items considerably exceeded the thresholds of .70 and .50, respectively [73]. These results indicate that each item relates strongly to the latent variable that it is assumed to measure.

Discriminant validity was assessed by checking intercorrelation coefficients, comparing the square root of AVE with the intercorrelation coefficients, and comparing loadings and cross-loadings [73,77,81]. Multimedia Appendix 18 shows that the intercorrelation coefficients (off-diagonal values) are located within acceptable ranges (<.85) [84]. Furthermore, each value of the square root of AVE for a construct (values on the diagonal) is higher than all intercorrelation coefficients between that construct and each other construct (Multimedia Appendix 18). As shown in Multimedia Appendix 19, the loading of each item on its construct was higher than the cross-loadings in rows and columns. The results of the three measures indicate that items of each construct are not related to the other constructs that it is not supposed to measure; therefore, the measurement model has acceptable discriminant validity.

### Structural Model

#### Model Fit and Predictive Power

**Figure 2:**
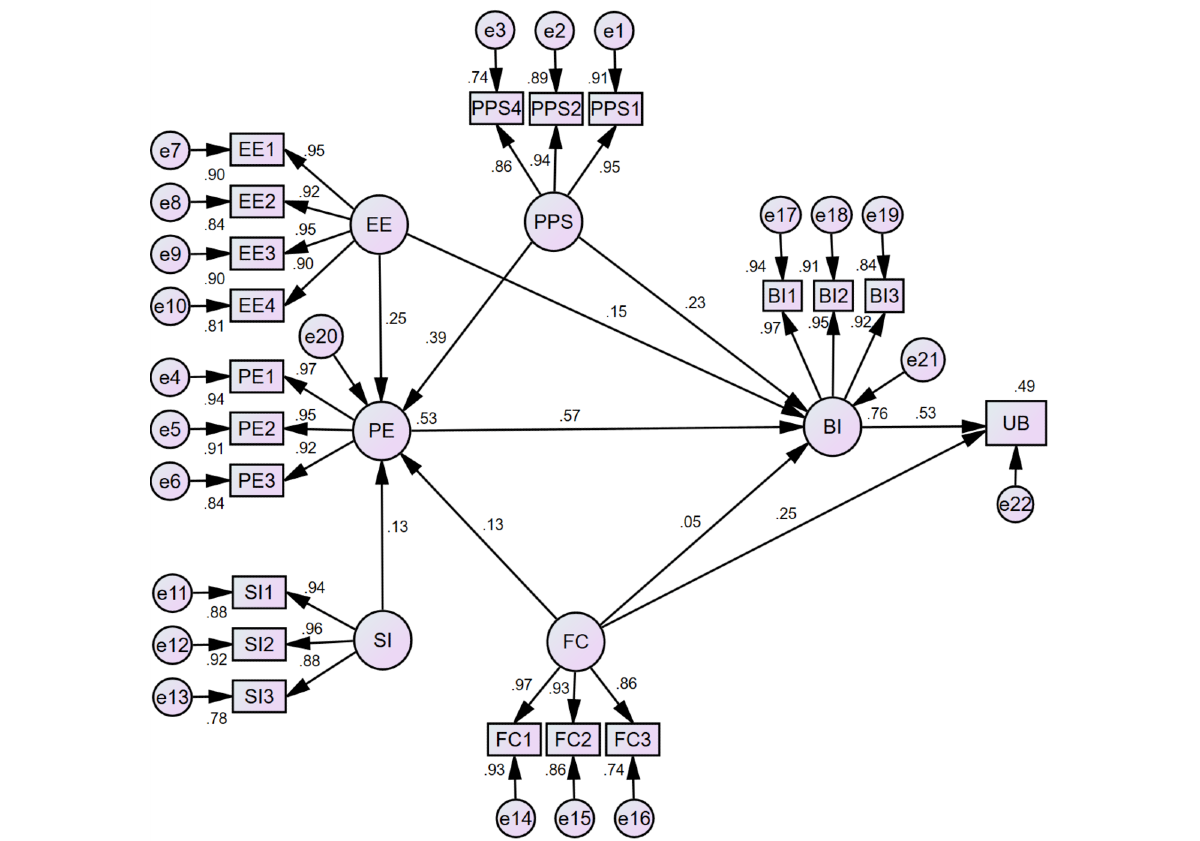
Structural model estimates.

The indices that were used to assess the fit of the measurement model were used again to assess the fit of the structural model. As shown in Multimedia Appendix 20, all fit indices were within the recommended values, indicating that the structural model adequately fits the collected data. The model was able to predict about 0.53 of the variance in PE, 0.76 of the variance in BI, and 0.49 of the variance in UB (Figure 2).

#### Strengths of Relationships

##### Direct Effects

As seen in Figure 2 and detailed in Table 2, all proposed direct effects were statistically significant, except for the effect of FCs on BI (β=.05, *P*=.08). Specifically, PPS was significantly associated with PE (β=.39) and BI (β=.23). The paths from EE to PE and BI were statistically significant (β=.25 and β=.15, respectively). There was a statistically significant relationship between SI and PE (β=.13). FCs were significantly associated with PE (β=.13) and UB (β=.25). The relationship between PE and BI was statistically significant (β=.57). BI and UB were significantly associated (β=.53). To sum up, the following hypotheses were supported: H1, H2, H7, H8, H13, H17, H19, H26, and H28 (Table 2).

**Table 2:** Results of the direct effects.

Hypothesis #	Path	Standardized estimate (β)	95% CI	*P* value
H1	PPS^a^→PE^b^	.39	0.32 to 0.46	<.001
H2	PPS→BI^c^	.23	0.17 to 0.29	<.001
H7	EE^d^→PE	.25	0.18 to 0.32	<.001
H8	EE→BI	.15	0.10 to 0.21	<.001
H13	SI^e^→PE	.13	0.04 to 0.22	<.001
H17	FCs^f^→PE	.13	0.06 to 0.20	<.001
H18	FCs→BI	.05	−0.003 to 0.10	.08
H19	FCs→UB^g^	.25	0.20 to 0.30	<.001
H26	PE→BI	.57	0.51 to 0.64	<.001
H28	BI→UB	.53	0.47 to 0.58	<.001

^a^PPS: perceived privacy and security.

^b^PE: performance expectancy.

^c^BI: behavioral intention.

^d^EE: effort expectancy.

^e^SI: social influence.

^f^FC: facilitating condition.

^g^UB: use behavior.

##### Mediating Effects

With regard to the mediating effects, results showed that PE significantly mediated the effect of PPS, EE, SI, and FCs on BI (β=.22, β=.14, β=.09, and β=.07, respectively; Table 3). Accordingly, H3, H9, H14, and H20 were supported in this study.

**Table 3:** Results of the mediating effects.

Hypothesis #	Indirect effect	Standardized estimate (β)	95% CI	*P* value
H3	PPS^a^→PE^b^→BI^c^	.22	0.18-0.28	<.001
H9	EE^d^→PE→BI	.15	0.10-0.19	<.001
H14	SI^e^→PE→BI	.09	0.04-0.14	<.001
H20	FCs^f^→PE→BI	.07	0.03-0.11	.002

^a^PPS: perceived privacy and security.

^b^PE: performance expectancy.

^c^BI: behavioral intention.

^d^EE: effort expectancy.

^e^SI: social influence.

^f^FC: facilitating condition.

##### Moderating Effects

With respect to the moderating effects, the effect of EE and FCs on BI statistically increased with increasing age (*P*=.03, *P*<.001, respectively; Table 4). In contrast, the effect of PE on BI and the effect of BI on UB statistically decreased with increasing age (*P*<.001, for both moderating effects).

**Table 4:** Results of the moderating effect of age.

Hypothesis #	Interaction effect	Standardized estimate (β)	*P* value
H4	PPS^a^×age→PE^b^	.18	.66
H5	PPS×age→BI^c^	−.02	.25
H10	EE^d^×age→PE	.14	.22
H11	EE×age→BI	.05	.03
H15	SI^e^×age→PE	.03	.45
H21	FCs^f^×age→PE	.21	.30
H22	FCs×age→BI	.03	.10
H23	FCs×age→UB^g^	.16	<.001
H27	PE×age→BI	−.10	<.001
H29	BI×age→UB	−.21	<.001

^a^PPS: perceived privacy and security.

^b^PE: performance expectancy.

^c^BI: behavioral intention.

^d^EE: effort expectancy.

^e^SI: social influence.

^f^FC: facilitating condition.

^g^UB: use behavior.

Concerning the moderating effects of sex, the association between PE and BI was statistically stronger for men than for women (β=.59 vs β=.50, *P*=.004; Table 5). The path from BI to UB was statistically stronger for women than for men (β=.53 vs β=.03, *P*=.001).

**Table 5:** Results of the moderating effect of sex.

Hypothesis #	Hypothesized path	Men		Women		*P* value for chi-square difference test
		Standardized estimate (β)	*P* value	Standardized estimate (β)	*P* value	
H4	PPS^a^→PE^b^	.41	<.001	.32	<.001	.81
H5	PPS→BI^c^	.25	<.001	.21	<.001	.39
H10	EE^d^→PE	.22	<.001	.26	<.001	.12
H11	EE→BI	.17	<.01	.17	<.01	.19
H15	SI^e^→PE	.068	.30	.17	<.001	.09
H21	FCs^f^→PE	.08	.23	.16	<.01	.14
H22	FCs→BI	.05	.35	.04	.33	.86
H23	FCs→UB^g^	.34	<.001	.24	<.001	.32
H27	PE→BI	.59	<.001	.50	<.001	.004
H29	BI→UB	.29	<.001	.53	<.001	.001

^a^PPS: perceived privacy and security.

^b^PE: performance expectancy.

^c^BI: behavioral intention.

^d^EE: effort expectancy.

^e^SI: social influence.

^f^FC: facilitating condition.

^g^UB: use behavior.

In relation to the moderating effect of education (Tables 6-8), the association between EE and PE was statistically stronger for the “secondary school or lower” group than for the “bachelor or higher” group (β=.31 vs β=.01, *P*=.049). The association between EE and BI was statistically weaker for the “bachelor or higher” group than for the “secondary school or lower” group (β=−.08 vs β=.13, *P*=.04) and for the college group (β=−.08 vs β=.12, *P*=.02; Tables 6-8). The path from FCs to UB was statistically stronger for the “secondary school or lower” group than for the college group (β=.38 vs β=.29, *P*=.003) and the “bachelor or higher” group (β=.38 vs β=.21, *P*=.03). The relationship between BI and UB was statistically stronger for the “bachelor or higher” group than for the “secondary school or lower” group (β=.48 vs β=.14, *P*<.001) and the college group (β=.48 vs β=.39, *P*=.003). The relationship between BI and UB was statistically stronger for the college group than for the “secondary school or lower” group (β=.39 vs β=.14, *P*<.001).

**Table 6:** Results of the moderating effect of education level (secondary school vs college).

Hypothesis #	Hypothesized path	Secondary school or lower		College or diploma		*P* value for chi-square difference test
		Standardized estimate (β)	*P* value	Standardized estimate (β)	*P* value	
H4	PPS^a^→PE^b^	.22	<.001	.25	<.001	.41
H5	PPS→BI^c^	.15	.01	.28	<.001	.18
H10	EE^d^→PE	.31	<.001	.14	.045	.93
H11	EE→BI	.13	.02	.12	.008	.43
H15	SI^e^→PE	.10	.13	.14	.047	.42
H21	FCs^f^→PE	.20	.004	.07	.31	.56
H22	FCs→BI	.05	.40	.02	.64	.82
H23	FCs→UB^g^	.38	<.001	.29	<.001	.003
H27	PE→BI	.55	<.001	.62	<.001	.53
H29	BI→UB	.14	.04	.39	<.001	.001

^a^PPS: perceived privacy and security.

^b^PE: performance expectancy.

^c^BI: behavioral intention.

^d^EE: effort expectancy.

^e^SI: social influence.

^f^FC: facilitating condition.

^g^UB: use behavior.

**Table 7:** Results of the moderating effect of education level (secondary school vs bachelor or higher).

Hypothesis #	Hypothesized path	Secondary school or lower		Bachelor or higher		*P* value for chi-square difference test
		Standardized estimate (β)	*P* value	Standardized estimate (β)	*P* value	
H4	PPS^a^→PE^b^	.22	<.001	.18	.03	.98
H5	PPS→BI^c^	.15	.01	.28	<.001	.07
H10	EE^d^→PE	.31	<.001	.01	.89	.049
H11	EE→BI	.13	.02	−.08	.20	.04
H15	SI^e^→PE	.10	.13	.17	.02	.37
H21	FCs^f^→PE	.20	.004	.18	.02	.27
H22	FCs→BI	.05	.40	.004	.95	.82
H23	FCs→UB^g^	.38	<.001	.21	.002	.03
H27	PE→BI	.55	<.001	.59	<.001	.24
H29	BI→UB	.14	.04	.48	<.001	.001

^a^PPS: perceived privacy and security.

^b^PE: performance expectancy.

^c^BI: behavioral intention.

^d^EE: effort expectancy.

^e^SI: social influence.

^f^FC: facilitating condition.

^g^UB: use behavior.

**Table 8:** Results of the moderating effect of education level (college vs bachelor or higher).

Hypothesis #	Hypothesized path	College or diploma		Bachelor or higher		*P* value for chi-square difference test
		Standardized estimate (β)	*P* value	Standardized estimate (β)	*P* value	
H4	PPS^a^→PE^b^	.25	<.001	.18	.03	.12
H5	PPS→BI^c^	.28	<.001	.28	<.001	.19
H10	EE^d^→PE	.14	.045	.01	.89	.17
H11	EE→BI	.12	.008	−.08	.20	.02
H15	SI^e^→PE	.14	.047	.17	.02	>.99
H21	FCs^f^→PE	.07	.31	.18	.02	.17
H22	FCs→BI	.02	.64	.004	.95	.91
H23	FCs→UB^g^	.29	<.001	.21	.002	.26
H27	PE→BI	.62	<.001	.59	<.001	.06
H29	BI→UB	.39	<.001	.48	<.001	.003

^a^PPS: perceived privacy and security.

^b^PE: performance expectancy.

^c^BI: behavioral intention.

^d^EE: effort expectancy.

^e^SI: social influence.

^f^FC: facilitating condition.

^g^UB: use behavior.

As shown in Tables 9-11, the association between FCs and UB was statistically stronger for patients with low-income than for patients with moderate income (β=.42 vs β=.23, *P*=.04) and higher income (β=.42 vs β=.07, *P*=.03). The path between FCs and UB was statistically stronger for patients with moderate income and those with high income (β=.23 vs β=.07, *P*=.003). The relationship between BI and UB was statistically stronger for patients with high income than for those with low income (β=.61 vs β=.43, *P*=.008) and middle income (β=.61 vs β=.41, *P*=.03).

**Table 9:** Results of the moderating effect of income (low income vs middle income).

Hypothesis #	Hypothesized path	Low income^a^		Middle income^b^			*P* value for chi-square difference test	
		Standardized estimate (β)	*P* value		Standardized estimate (β)	*P* value		
H4	PPS^c^→PE^d^	.38	<.001		.40	<.001		.71
H5	PPS→BI^e^	.24	<.001		.27	<.001		.91
H10	EE^f^→PE	.18	<.001		.32	<.001		.07
H11	EE→BI	.14	<.001		.21	<.001		.41
H15	SI^g^→PE	.14	.006		.13	.07		.98
H21	FCs^h^→PE	.22	<.001		.06	.40		.43
H22	FCs→BI	.08	.09		.06	.31		.96
H23	FCs→UB^i^	.42	<.001		.23	<.001		.04
H27	PE→BI	.53	<.001		.52	<.001		.40
H29	BI→UB	.43	<.001		.41	<.001		.87

^a^Low income: <US $25,000.

^b^Medium income: US $25,000-US $50,999.

^c^PPS: perceived privacy and security.

^d^PE: performance expectancy.

^e^BI: behavioral intention.

^f^EE: effort expectancy.

^g^SI: social influence.

^h^FC: facilitating condition.

^i^UB: use behavior.

**Table 10:** Results of the moderating effect of income (low income vs high income).

Hypothesis #	Hypothesized path	Low income^a^		High income^b^		*P* value for chi-square difference test
		Standardized estimate (β)	*P* value	Standardized estimate (β)	*P* value	
H4	PPS^c^→PE^d^	.38	<.001	.39	<.001	.92
H5	PPS→BI^e^	.24	<.001	.23	<.001	.81
H10	EE^f^→PE	.18	<.001	.24	.01	.60
H11	EE→BI	.14	<.001	.09	.13	.53
H15	SI^g^→PE	.14	.006	.19	.054	.45
H21	FCs^h^→PE	.22	<.001	−.11	.29	.06
H22	FCs→BI	.08	.09	.03	.63	.96
H23	FCs→UB^i^	.42	<.001	.07	.40	.03
H27	PE→BI	.53	<.001	.66	<.001	.12
H29	BI→UB	.43	<.001	.61	<.001	.008

^a^Low income: <US $25,000.

^b^High income: ≥US $51,000.

^c^PPS: perceived privacy and security.

^d^PE: performance expectancy.

^e^BI: behavioral intention.

^f^EE: effort expectancy.

^g^SI: social influence.

^h^FC: facilitating condition.

^i^UB: use behavior.

**Table 11:** Results of the moderating effect of income (middle income vs high income).

Hypothesis #	Hypothesized path	Middle income^a^		High income^b^		*P* value for chi-square difference test	
		Standardized estimate (β)	*P* value	Standardized estimate (β)	*P* value		
H4	PPS^c^→PE^d^	.40	<.001	.39	<.001		.83
H5	PPS→BI^e^	.27	<.001	.23	<.001		.75
H10	EE^f^→PE	.32	<.001	.24	.01		.22
H11	EE→BI	.21	<.001	.09	.13		.21
H15	SI^g^→PE	.13	.07	.19	.054		.51
H21	FCs^h^→PE	.06	.40	−.11	.29		.18
H22	FCs→BI	.06	.31	.03	.63		.96
H23	FCs→UB^i^	.23	<.001	.07	.40		.003
H27	PE→BI	.52	<.001	.66	<.001		.07
H29	BI→UB	.41	<.001	.61	<.001		.03

^a^Medium income: US $25,000-US $50,999.

^b^High income: ≥US $51,000.

^c^PPS: perceived privacy and security.

^d^PE: performance expectancy.

^e^BI: behavioral intention.

^f^EE: effort expectancy.

^g^SI: social influence.

^h^FC: facilitating condition.

^i^UB: use behavior.

With respect to the moderating effect of internet access (Table 12), the paths EE→BI and FCs→UB were statistically stronger for patients without internet access than for those with internet access (*P*=.03, *P*<.001, respectively). In contrast, the paths PE→BI and BI→UB were statistically stronger for patients with internet access than for those without internet access (*P*=.005 and *P*=.002, respectively). According to the results of all moderating effects, the following hypotheses were partially supported: H10, H11, H23, H27, and H29.

**Table 12:** Results of the moderating effect of internet access.

Hypothesis #	Hypothesized path	Internet access		No internet access			*P* value for chi-square difference test	
		Standardized estimate (β)	*P* value		Standardized estimate (β)	*P* value		
H4	PPS^a^→PE^b^	.40	<.001		.35	<.001		.68
H5	PPS→BI^c^	.21	<.001		.30	.002		.93
H10	EE^d^→PE	.25	<.001		.24	.02		.36
H11	EE→BI	.10	<.001		.27	.007		.03
H15	SI^e^→PE	.14	<.001		.07	.51		.26
H21	FCs^f^→PE	.12	.006		.22	.06		.89
H22	FCs→BI	.04	.17		.07	.46		.89
H23	FCs→UB^g^	.20	.03		.34	<.001		<.001
H27	PE→BI	.59	<.001		.39	<.001		.005
H29	BI→UB	.51	<.001		.31	.004		.002

^a^PPS: perceived privacy and security.

^b^PE: performance expectancy.

^c^BI: behavioral intention.

^d^EE: effort expectancy.

^e^SI: social influence.

^f^FCs: facilitating conditions.

^g^UB: use behavior.

##### Moderated Mediating Effects

With regard to the proposed moderated mediations, the indirect effects of EE and SI on BI were statistically stronger for women than for men (*P*=.03 and *P*=.01, respectively; Table 13). The indirect effect of PPS on BI was stronger for patients with college or diploma compared with those with secondary school and lower (Tables 14-16). In contrast, the indirect effect of EE on BI was stronger for patients with secondary school or lower than for those with college or diploma (Tables 14-16). There was no moderating effect of income on all indirect effects (Tables 16-19). As shown in Table 20, the indirect effect of PPS on BI is statistically stronger for patients with internet access (*P*<.001). The indirect effect of EE on BI was statistically stronger for patients without internet access (*P*=.03). Accordingly, the following hypotheses were partially supported: H6, H12, and H16.

**Table 13:** Results of the moderating effect of sex on indirect paths.

Hypothesis #	Hypothesized path	Men		Women			*P* value for chi-square difference test	
		Standardized estimate (β)	*P* value		Standardized estimate (β)	*P* value		
H6	PPS^a^→PE^b^→BI^c^	.21	<.001		.19	<.001		.24
H12	EE^d^→PE→BI	.11	<.001		.16	<.001		.03
H16	SI^e^→PE→BI	.03	.18		.10	<.001		.01
H24	FCs^f^→PE→BI	.04	.23		.10	.004		.06

^a^PPS: perceived privacy and security.

^b^PE: performance expectancy.

^c^BI: behavioral intention.

^d^EE: effort expectancy.

^e^SI: social influence.

^f^FC: facilitating condition.

**Table 14:** Results of the moderating effect of education on indirect paths (school vs college).

Hypothesis #	Hypothesized path	Secondary school or lower		College or diploma		*P* value for chi-square difference test
		Standardized estimate (β)	*P* value	Standardized estimate (β)	*P* value	
H6	PPS^a^→PE^b^→BI^c^	.12	<.001	.30	.002	.007
H12	EE^d^→PE→BI	.17	<.001	.01	.90	.045
H16	SI^e^→PE→BI	.06	.11	.08	.02	.45
H24	FCs^f^→PE→BI	.11	.007	.04	.31	.49

^a^PPS: perceived privacy and security.

^b^PE: performance expectancy.

^c^BI: behavioral intention.

^d^EE: effort expectancy.

^e^SI: social influence.

^f^FC: facilitating condition.

**Table 15:** Results of the moderating effect of education on indirect paths (school vs bachelor).

Hypothesis #	Hypothesized path	Secondary school or lower		Bachelor or higher		*P* value for chi-square difference test
		Standardized estimate (β)	*P* value	Standardized estimate (β)	*P* value	
H6	PPS^a^→PE^b^→BI^c^	.12	<.001	.11	.04	.75
H12	EE^d^→PE→BI	.17	<.001	.08	.09	.81
H16	SI^e^→PE→BI	.06	.11	.10	.03	.27
H24	FCs^f^→PE→BI	.11	.007	.10	.05	.26

^a^PPS: perceived privacy and security.

^b^PE: performance expectancy.

^c^BI: behavioral intention.

^d^EE: effort expectancy.

^e^SI: social influence.

^f^FC: facilitating condition.

**Table 16:** Results of the moderating effect of education on indirect paths (college vs bachelor).

Hypothesis #	Hypothesized path	College or diploma		Bachelor or higher			*P* value for chi-square difference test	
		Standardized estimate (β)	*P* value		Standardized estimate (β)	*P* value		
H6	PPS^a^→PE^b^→BI^c^	.30	.002		.11	.04		.16
H12	EE^d^→PE→BI	.01	.90		.08	.09		.16
H16	SI^e^→PE→BI	.08	.02		.10	.03		.59
H24	FCs^f^→PE→BI	.04	.31		.10	.05		.14

^a^PPS: perceived privacy and security.

^b^PE: performance expectancy.

^c^BI: behavioral intention.

^d^EE: effort expectancy.

^e^SI: social influence.

^f^FC: facilitating condition.

**Table 17:** Results of the moderating effect of income on indirect paths (low income vs middle income).

Hypothesis #	Hypothesized path	Low income		Middle income		*P* value for chi-square difference test
		Standardized estimate (β)	*P* value	Standardized estimate (β)	*P* value	
H6	PPS^a^→PE^b^→BI^c^	.20	<.001	.21	<.001	.84
H12	EE^d^→PE→BI	.10	<.001	.19	.002	.13
H16	SI^e^→PE→BI	.07	.01	.07	.09	.90
H24	FCs^f^→PE→BI	.12	.002	.03	.37	.32

^a^PPS: perceived privacy and security.

^b^PE: performance expectancy.

^c^BI: behavioral intention.

^d^EE: effort expectancy.

^e^SI: social influence.

^f^FC: facilitating condition.

**Table 18:** Results of the moderating effect of income on indirect paths (low income vs high income).

Hypothesis #	Hypothesized path	Low income		High income		*P* value for chi-square difference test
		Standardized estimate (β)	*P* value	Standardized estimate (β)	*P* value	
H6	PPS^a^→PE^b^→BI^c^	.20	<.001	.27	.002	.56
H12	EE^d^→PE→BI	.10	<.001	.16	.03	.37
H16	SI^e^→PE→BI	.07	.01	.13	.04	.28
H24	FCs^f^→PE→BI	.12	.002	−.07	.22	.06

^a^PPS: perceived privacy and security.

^b^PE: performance expectancy.

^c^BI: behavioral intention.

^d^EE: effort expectancy.

^e^SI: social influence.

^f^FC: facilitating condition.

**Table 19:** Results of the moderating effect of income on indirect paths (middle income vs high income).

Hypothesis #	Hypothesized path	Middle income		High income			*P* value for chi-square difference test	
		Standardized estimate (β)	*P* value		Standardized estimate (β)	*P* value		
H6	PPS^a^→PE^b^→BI^c^	.21	<.001		.27	.002		.45
H12	EE^d^→PE→BI	.19	.002		.16	.03		.84
H16	SI^e^→PE→BI	.07	.09		.13	.04		.30
H24	FCs^f^→PE→BI	.03	.37		−.07	.22		.17

^a^PPS: perceived privacy and security.

^b^PE: performance expectancy.

^c^BI: behavioral intention.

^d^EE: effort expectancy.

^e^SI: social influence.

^f^FC: facilitating condition.

**Table 20:** Results of the moderating effect of internet access on indirect paths.

Hypothesis #	Hypothesized path	Internet access		No internet access		*P* value for chi-square difference test
		Standardized estimate (β)	*P* value	Standardized estimate (β)	*P* value	
H6	PPS^a^→PE^b^→BI^c^	.25	<.001	.02	.72	.001
H12	EE^d^→PE→BI	.10	.02	.15	<.001	.03
H16	SI^e^→PE→BI	.08	<.001	.03	.42	.06
H24	FCs^f^→PE→BI	.07	.004	.09	.04	.54

^a^PPS: perceived privacy and security.

^b^PE: performance expectancy.

^c^BI: behavioral intention.

^d^EE: effort expectancy.

^e^SI: social influence.

^f^FC: facilitating condition.

## Discussion

### Principal Findings

This study aimed to improve the predictive power of a model proposed by Abd-Alrazaq et al [38] by proposing and examining new relationships between the variables existing in that model. The predictive power of the new model was slightly higher than that of the Abd-Alrazaq model for PE (53% vs 51%) and UB (49% vs 48%), but it was exactly the same in both models for BI (76%).

With regard to the direct effects, there was no considerable difference between the new model and the Abd-Alrazaq model for the following paths PE→BI (0.57 vs 0.57), EE→BI (0.15 vs 0.16), PPS→BI (0.23 vs 0.24), FCs→UB (0.25 vs 0.25), and BI→UB (0.53 vs 0.53). Compared with the Abd-Alrazaq model, the current model showed a considerable decrease in the effect of EE (0.25 vs 0.34) and PPS (0.39 vs 0.49) on PE; however, both paths were still significant in the current model. This decrease resulted from proposing 2 new predictors for PE (ie, SI and FCs) in the current model, which were significant. The only direct path that was nonsignificant was FCs→BI in the current model. This finding is in line with the findings of a study conducted by Tavares and Oliveira, who did not find a significant association between FCs and BI to use ePHRs [40]. Venkatsh et al [39] attributed this nonsignificant path to the fact that this effect disappears when a model includes both PE and EE.

Compared with the Abd-Alrazaq model, the current model showed a decrease in the indirect associations between BI and each of EE (0.15 vs 0.20) and PPS (0.22 vs 0.28) through PE. However, both indirect effects are still significant in the current model. This decrease resulted from proposing 2 new predictors for PE (ie, SI and FCs) in the current model. Two new indirect paths were found SI→PE→BI and FCs→PE→BI. This means that patients who perceive that important others believe they should use GP online services are more likely to perceive it as a useful system; therefore, they are more likely to intend to use it. Furthermore, patients who believe that an organizational and technical infrastructure exists to support the use of GP online services are more likely to perceive it as a useful system and are therefore more likely to intend to use it.

All proposed moderating effects that are common between the new model and the Abd-Alrazaq model were comparable between both models. In addition to the significant moderating effects found in the Abd-Alrazaq model, this study found that the association between BI and UB is significantly moderated by age, sex, education, income, and internet access and that the association between EE and PE is moderated by education. Specifically, the association between BI and UB is stronger for younger women with higher levels of education, income, and internet access, and the association between EE and PE is stronger for patients with lower levels of education.

With regard to the moderated mediations proposed in the new model, this study found that the indirect effect of EE on BI through PE was statistically stronger for women without internet access. The indirect effect of PPS on BI was stronger for patients with college education or diploma compared with those with secondary school education and lower, whereas the indirect effect of EE on BI was stronger for patients with secondary school or lower than for those with college education or diplomas. Furthermore, the indirect effect of SI on BI through PE was stronger for patients without internet access. Last but not least, the indirect effect of PPS on BI through PE was statistically stronger for patients with internet access.

### Theoretical and Practical Contributions

This study is one of the very few theory-based studies conducted to identify the factors that affect patients’ use of ePHRs or patient portals [21,25-27]. The predictive power of the new model (49%) is higher than that of the previous models proposed in our previous study (48%) and other studies conducted in the context of ePHRs: Tavares and Oliveira (26.8%) [40] and Hsieh (42.7%) [85]. Moreover, the predictive power of the new model is higher than that of the original UTAUT model (48%) [39]. Accordingly, this study contributes to the literature by providing the most predictive model to explain the adoption of ePHRs to date.

To the best of our knowledge, this is the first study in the context of ePHRs that examined the direct effect of SI and FCs on PE, their indirect effects on BI through PE, the moderation effects on the association between BI and UB, and the moderated effects on indirect relationships. This extends our understanding of the complex associations between the factors that affect the adoption of ePHRs.

In addition to the practical contributions reported in the previous study [38], this study provides some contributions based on the newly proposed relationships. People who are important to patients (eg, family members, friends, physicians, and caregivers) can play an important role in enhancing their perceived usefulness of the system and their intention to use it. The influence of these important people is more evident on women than on men. Interventions aimed at increasing the uptake of online access could harness the influence of such individuals to encourage patients to use such services. For example, GPs could prompt the recurrent users of the system to become ePHR champions and speak to their friends or family members about their experiences. GPs could also train practice staff to offer these services to their patients routinely in their communications, and campaigns aimed at increasing ePHR uptake could use social influencing techniques, such as celebrity endorsements. As FCs are directly associated with perceived usefulness of ePHRs, and this, in turn affects BIs, steps should be taken to improve the degree to which patients believe that an organizational and technical infrastructure exists to support their use. For example, the National Health Service app has demonstrated an efficient infrastructure supporting the patient registration process, which enables patients to sign up to access their records online without needing to visit their GP surgery [15]. Instead of registration requiring patients to show evidence of their identity to practice staff, they can instead register by uploading a photograph of identifying documentation and taking a short selfie video on their mobile device. Other potential approaches to targeting FCs include the provision of online educational materials, 24/7 technical support, or drop-in training sessions at GP practices.

### Research Limitations

The proposed model was examined using data collected from 4 GP practices that have implemented the same system (SystmOnline); therefore, our findings may not be generalizable to other systems (eg, Patient Access and i-Patient). Nonetheless, the findings may still be applicable to other ePHRs because all participants were nonusers, and these systems offer the same services to the patients. Consequently, participants would be unlikely to have different perceptions of the different systems.

This study focused on the factors that influence the initial use of ePHRs, given that the system is new in England and has a low adoption rate. Thus, the generalizability of the findings in the context of the continuing use of ePHRs is limited. Given that the study used secondary data, it was not possible to assess the effect of new factors, such as those recommended by Abd-Alrazaq et al [38].

Sampling bias may be a concern in this study owing to the convenience sampling technique used to recruit the participants [37,86]. This study showed that there was no statistically significant difference between the participants and nonparticipants in terms of age, sex, and ethnicity. Accordingly, our findings may be generalizable to GPs, similar to the 4 GPs in this study.

### Recommendations for Future Research

The applicability of the proposed model to other contexts should be examined in further studies. Specifically, researchers may assess the applicability of the model to other providers of GP online services (eg, Patient Access), specific platforms (eg, mobiles, tablets, and computers), other settings (eg, hospitals), and other cities or countries.

Further studies are required to validate the new significant associations proposed in this study, such as SI→PE, FCs→PE, and SI→PE→BI. In addition, future studies should endeavor to improve the predictive power of the current model by adding other factors such as awareness of the system, health status, perceived severity, patient satisfaction, and patient activation level.

It is well known that the eventual success of information technology depends on continued use more than initial use [33,87-89]. There is a lack of studies that have assessed factors affecting the continuing use of ePHRs or even consumer health information technologies (CHITs). Therefore, we prompt researchers to develop and examine a theoretical model that explains the variables affecting the continuing use of ePHRs and CHITs.

This study did not assess series mediations, such as the indirect effect of EE on UB through PE and BI (ie, EE→PE→BI→UB). Furthermore, to the best of our knowledge, such effects have not been examined in previous studies in the context of ePHRs and CHITs. This highlights a need to assess such effects.

### Conclusions

This study slightly improved the predictive power of the Abd-Alrazaq model. More importantly, the improved model showed new significant relationships that were not examined before in the ePHR context, such as the direct effect of SI and FCs on PE, their indirect effects on BI through PE, moderation effect of age, sex, educational level, income, and internet access on the association between BI and UB, and the moderating effects on some indirect relationships. These findings extend our understanding of the complex associations between factors affecting the adoption of ePHRs. The predictive power of 49% indicates that there are other, as yet unidentified, factors that affect the use of ePHRs. Further studies are required to validate the new model in different contexts and to improve its predictive power by proposing new factors. Interventions could focus on the role of significant others (eg, health care professionals, friends, and family members) in influencing web access usage, for example, by discussing the potential benefits of such services with patients.

## Appendix

Multimedia Appendix 1 Selection of theory.

Multimedia Appendix 2 The previous model.

Multimedia Appendix 3 Justification of adaptation of UTAUT.

Multimedia Appendix 4 Conceptual definitions of constructs.

Multimedia Appendix 5 Characteristics of the four general practices.

Multimedia Appendix 6 Measures of constructs.

Multimedia Appendix 7 Questionnaire.

Multimedia Appendix 8 Participants' characteristics.

Multimedia Appendix 9 Histograms.

Multimedia Appendix 10 Values of skewness and kurtosis.

Multimedia Appendix 11 Scatterplot graphs.

Multimedia Appendix 12 Curve estimation procedure.

Multimedia Appendix 13 Tolerance values.

Multimedia Appendix 14 Harman’s single-factor test.

Multimedia Appendix 15 Results of fit indices of the measurement model.

Multimedia Appendix 16 Results of construct reliability.

Multimedia Appendix 17 Results of convergent validity.

Multimedia Appendix 18 Intercorrelation coefficients and squared roots of AVE.

Multimedia Appendix 19 Item loadings and cross-loadings.

Multimedia Appendix 20 Results of fit indices of the structural model.

## Abbreviations

AVE: average variance extracted
BI: behavioral intention
CHIT: consumer health information technology
EE: effort expectancy
ePHR: electronic personal health record
FC: facilitating condition
GP: general practice
H: hypothesis
PE: performance expectancy
PPS: perceived privacy and security
SEM: structural equation modeling
SI: social influence
UB: use behavior
UTAUT: Unified Theory of Acceptance and Use of Technology
