# Supplementary material for: Patients’ Adoption of Electronic Personal Health Records in England: Secondary Data Analysis
Source: J Med Internet Res. 2020 Oct 7;22(10):e17499. doi: 10.2196/17499 (PMC7578819; doi:10.2196/17499)
Supplement: Multimedia Appendix 1 [file jmir_v22i10e17499_app1.docx]

Appendix 1: Selection of Theory

After reviewing literature in various disciplines (e.g. psychology, sociology, and information systems), the following theories and models were identified to be suitable in the context of ePHRs: Innovation Diffusion Theory (IDT), Theory of Reasoned Action (TRA), Theory of Planned Behaviour (TPB), Social Cognitive Theory (SCT), Technology Acceptance Model (TAM), Technology Acceptance Model 2 (TAM 2), Technology Acceptance Model 3 (TAM 3), Model of Personal Computer Utilisation (MPCU), Motivational Model (MM), Combined TAM and TBP model (C-TAM-TPB), Unified Theory of Acceptance and Use of Technology (UTAUT), Unified Theory of Acceptance and Use of Technology 2 (UTAUT 2). In order to be objective in selecting the most appropriate theory or model, the current study identified six criteria for selecting an appropriate theory based on recommendations by Bhattacherjee [1] and Taylor and Todd [2]. While two criteria are related to the applicability of the theory on the phenomena of interest (i.e. population and type of behaviour), the remaining four criteria are related to goodness of the theory (i.e. logical consistency, explanatory power, falsifiability, and parsimony).

The first criterion is the applicability of the theory to the population of interest [2]. The population in the present study is healthcare consumers (i.e. patients), therefore, the theory must be suitable for understanding consumer behaviour rather than other populations such as employees. The second criterion is the applicability of the theory to the behaviour under investigation [2]. The behaviour of interest in the present study is voluntary (i.e. ePHRs use), thus, the theory must be appropriate for studying voluntary behaviours but not compulsory behaviours. The third criterion is logical consistency, which refers to the degree to which proposed relationships are rational and logical [1]. The fourth criterion is explanatory power, which refers to the ability of theory to account for reality [1]. The fifth criterion is falsifiability, which refers to the possibility of disproving the theory through empirical tests (i.e. theory must have adequate explanations and measurable constructs) [1]. The sixth criterion is parsimony, which refers to the ability of theory to examine a phenomenon using few numbers of variables [1]. As shown in Table 1, UTAUT was the only theory that met all those criteria. Therefore, this study chose UTAUT as a theoretical lens to study factors that affect patients’ use of ePHRs. More details about how the theories met or did not meet each criterion are discussed below.

**Table 1: Criteria of Selection of Theory**


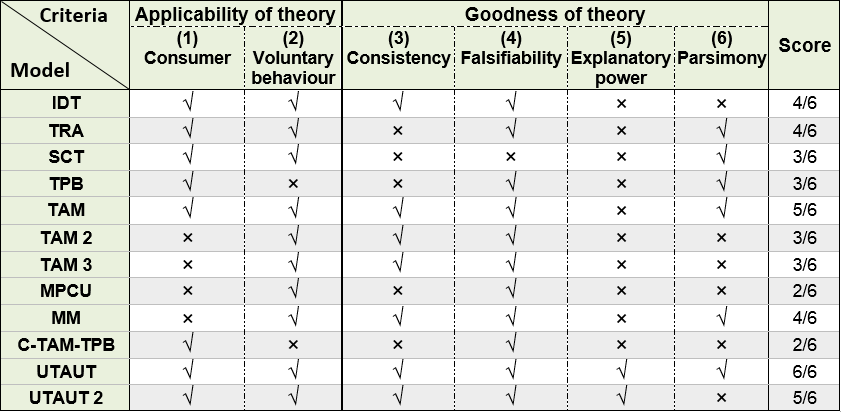


Starting with the first criterion (population), the following theories include constructs (e.g. job fit and output quality) that make these theories more suitable for explaining employees’ behaviours; TAM 2, TAM 3, MPCU, and MM. Other theories do not consider the difference between the behaviour of employees and consumers; IDT, TRA, SCT, TPB, TAM, and C-TAM-TPB. However, they have been used widely used for explaining consumer adoption behaviour [e.g. 3, 4-7]. One theory (UTAUT) aimed to understand the adoption of technology among employees. However, Venkatesh, Thong [8] confirmed that UTAUT can be suitable for investigating technology adoption among consumers as they demonstrated that UTAUT was able to account for 56% and 40% of the variance in behavioural intention and use behaviour, respectively. Lastly, UTAUT 2 was the only theory that was developed to understand technology adoption among consumers. Accordingly, only the following theories did not meet the first criterion as they include constructs suitable only for explaining employees’ behaviours: TAM 2, TAM 3, MPCU, and MM.

With respect to the second criterion (type of behaviour), five theories are appropriate for understanding voluntary and non-voluntary behaviours; IDT, SCT, TAM 2, TAM 3, UTAUT. On the other hand, five theories are applicable only in voluntary contexts; TRA, TAM, MPCU, MM, and UTAUT 2. In contrast, TPB and C-TAM-TPB are more suitable for non-voluntary behaviours. Thus, all theories met this criterion, bar TPB and C-TAM-TPB (see Table 3.1).

With reference to the third criterion (logical consistency), TRA was criticised because it does not posit any relationship between subjective norms and attitudes while several studies found that social norms affect attitudes such as Greene, Hale [9], Park [10], and Shepherd and O'keefe [11]. Besides, one of TRA’s flaws is its assumption that attitude and subjective norms are the only predictors of a voluntary behaviour, and other external factors may affect the behaviour indirectly only through attitude and subjective norms [12, 13]. In SCT and MPCU, the behavioural intention was ignored although it has been considered as an essential part of most human behaviour theories [14-17]. The logical consistency of C-TAM-TPB is also weak since it posits that though some relationships were not significant (e.g. the relationship between perceived ease of use and attitude), they included in the model [18]. Accordingly, IDT, TAM, TAM 2, TAM 3, MM, UTAUT, and UTAUT 2 have a logical consistency (see Table 3.1).

All theories met the fourth criterion (i.e. falsifiability) except SCT. Precisely, as mentioned before, constructs in SCT are difficult to be operationalised because they cover an extensive range of factors that are not clear from the theory [16, 19]. Consequently, SCT is the only theory that did not meet the fourth criterion (see Table 3.1).

With regard to the explanatory power of the theory (the fifth criterion), most of the abovementioned theories were not validated by their authors to assess the explanatory power (e.g. IDT, TRA, SCT, and TPB). Since Venkatesh, Morris [17] tested nine theories in one study (IDT, TRA, SCT, TPB, TAM, TAM 2, MPCT, MM, and C-TAM-TPB), it would be fair to use results of Venkatesh’s study to compare nine theories in terms of the explanatory power. However, Venkatesh’s study did not examine the explanatory power of TAM 3 and UTAUT 2. Fortunately, authors of both theories validated them and, thereby, explanatory powers resulted from these studies will be used to compare the theories. In short, Venkatesh, Morris [17] found that all nine models were able to explain between 17% and 42% of the variance in behavioural intention, and between 35% and 39% of the variance in use behaviour. TAM 3 accounted for 53% and 35% of the variance in behavioural intention and use behaviour, respectively [20]. UTAUT was able to predict 70% of the variance in behavioural intention, and 48% of variance in use behaviour [17]. UTAUT 2 explained 74% and 52% of the variance in behavioural intention and use behaviour, respectively [8]. It is clear that UTAUT and UTAUT 2 have stronger explanatory power than other theories, thereby, they met the fifth criterion (see Table 3.1). As for the last criterion (parsimony), only six theories were considered parsimonious, which are: TRA, SCT, TPB, MM, TAM, and UTAUT [21-25].

In addition to that UTAUT met all criteria, it has the following pros. First, UTAUT does not focus only on human-technology factors (e.g. performance expectancy and effort expectancy) as some theories do (e.g. TAM), but it includes personal factors (e.g. age), organisational factors (facilitating conditions), and social factors (social influence) [26]. Second, UTAUT is one of few theories that use moderators in their model, and this enriches understanding of use behaviour of technology [17]. Last, UTAUT may be applicable to different technologies in different countries [27]. This is because it has been validated extensively in different fields and contexts, and it showed that it is a suitable theory for investigating technology acceptance [21, 26, 28, 29]. Further, it has been widely used by studies in the context of consumer health information technologies (CHITs) [e.g. 26, 28, 30, 31, 32].

**References**

1. Bhattacherjee A. Social science research: principles, methods, and practices 2012. Available from: <http://scholarcommons.usf.edu/>.

2. Taylor S, Todd P. Understanding information technology usage: A test of competing models. Information Systems Research. 1995;6(2):144-76.

3. Abramson EL, Patel V, Edwards A, Kaushal R. Consumer perspectives on personal health records: a 4-community study. The American Journal of Managed Care. 2014;20(4):287-98.

4. Baird A. Extending adoption of innovation theory with consumer influence the case of personal health records (PHRs) and patient portals: Arizona State University; 2012.

5. Emani S, Yamin CK, Peters E, Karson AS, Lipsitz SR, Wald JS, et al. Patient perceptions of a personal health record: a test of the diffusion of innovation model. Journal of medical Internet research. 2012;14(6):1-15. PMID: 23128775. doi: 10.2196/jmir.2278.

6. Lazard AJ, Watkins I, Mackert MS, Xie B, Stephens KK, Shalev H. Design simplicity influences patient portal use: the role of aesthetic evaluations for technology acceptance. Journal of the American Medical Informatics Association. 2016;23(e1):e157-e61. doi: 10.1093/jamia/ocv174.

7. Torres CA. Examining the role of anxiety and apathy in health consumers' intentions to use patient health portals for personal health information management. United States Florida State University; 2011.

8. Venkatesh V, Thong JY, Xu X. Consumer acceptance and use of information technology: extending the unified theory of acceptance and use of technology. Management Information Systems Quarterly. 2012;36(1):157-78

9. Greene K, Hale JL, Rubin DL. A test of the theory of reasoned action in the context of condom use and AIDS. Communication Reports. 1997;10(1):21-33.

10. Park HS. Relationships among attitudes and subjective norms: testing the theory of reasoned action across cultures. Communication Studies. 2000;51(2):162-75.

11. Shepherd GJ, O'keefe DJ. Separability of attitudinal and normative influences on behavioral intentions in the Fishbein-Ajzen model. The Journal of Social Psychology. 1984;122(2):287-8.

12. Davis FD. Perceived usefulness, perceived ease of use, and user acceptance of information technology. Management Information Systems Quarterly. 1989;13(3):319-40.

13. Hale JL, Householder BJ, Greene KL. The theory of reasoned action. In: Dillard JP, Pfau M, editors. The persuasion handbook: Developments in theory and practice. Thousand Oaks, USA: Sage; 2002.

14. Bandura A. Social foundations of thought and action: a social cognitive theory. Englewood Cliffs, NJ: Prentice Hall; 1986.

15. Chang, Cheung W. Determinants of the intention to use Internet/WWW at work: a confirmatory study. Information & Management. 2001;39(1):1-14.

16. Compeau D, Higgins CA. Computer self-efficacy: development of a measure and initial test. Management Information Systems Quarterly. 1995;19(2):189-211.

17. Venkatesh V, Morris MG, Davis GB, Davis FD. User acceptance of information technology: toward a unified view. Management Information Systems Quarterly. 2003;27(3):425-78.

18. Taylor S, Todd P. Assessing IT usage: the role of prior experience. Management Information Systems Quarterly. 1995;19(4):561-70.

19. Munro S, Lewin S, Swart T, Volmink J. A review of health behaviour theories: how useful are these for developing interventions to promote long-term medication adherence for TB and HIV/AIDS? BMC Public Health. 2007;7(1):1-16.

20. Venkatesh V, Bala H. Technology acceptance model 3 and a research agenda on interventions. Decision Sciences. 2008;39(2):273-315.

21. Assadi V. Adoption of integrated personal health record systems: a self-determination theory perspective: McMaster University; 2013.

22. Bagozzi RP. The legacy of the technology acceptance model and a proposal for a paradigm shift. Journal of the Association for Information Systems. 2007;8(4):244-54.

23. Gartrell K. Factors associated with electronic personal health record use among registered nurses for their own health management. Maryland, USA: University of Maryland; 2014.

24. Holden RJ, Karsh B. The technology acceptance model: its past and its future in health care. Journal of Biomedical Informatics. 2010;43(1):159-72.

25. Whetstone M, Goldsmith R. Factors influencing intention to use personal health records. International Journal of Pharmaceutical and Healthcare Marketing. 2009;3(1):8-25. doi: 10.1108/17506120910948485.

26. Or CKL. Development of a model of consumer health information technology acceptance of patients with chronic illness. USA: University of Wisconsin-Madison; 2008.

27. Or CKL, Karsh BT. A Systematic review of patient acceptance of consumer health information technology. Journal of the American Medical Informatics Association. 2009;16(4):550-60. doi: 10.1197/jamia.M2888.

28. Cimperman M, Brenčič MM, Trkman P. Analyzing older users’ home telehealth services acceptance behavior—applying an Extended UTAUT model. International journal of medical informatics. 2016;90(1):22-31.

29. Tavares J, Oliveira T. Electronic health record patient portal adoption by health care consumers: an acceptance model and survey. Journal of medical Internet research. 2016;18(3):1-19. doi: 10.2196/jmir.5069.

30. Beenkens FHC. Acceptance of e-health technology: a patient perspective: Delft University of Technology; 2011.

31. de Veer AJ, Peeters JM, Brabers AE, Schellevis FG, Rademakers JJJ, Francke AL. Determinants of the intention to use e-Health by community dwelling older people. BMC Health Services Research. 2015;15(1):1-9.

32. Mekawie NM. Factors affecting adoption of eHealth in Egypt: Middlesex University; 2013.
