# Supplementary figures and images for: Patients’ Adoption of Electronic Personal Health Records in England: Secondary Data Analysis
Source: J Med Internet Res. 2020 Oct 7;22(10):e17499. doi: 10.2196/17499 (PMC7578819; doi:10.2196/17499)

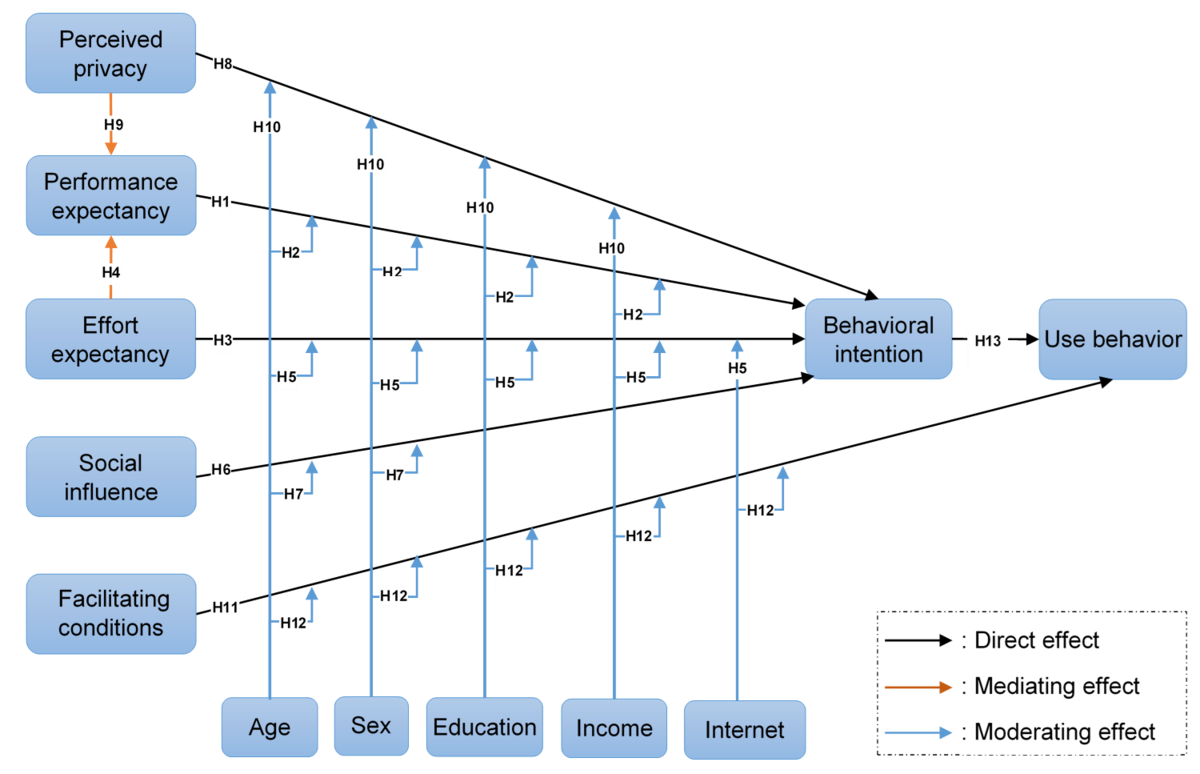

Supplement: Multimedia Appendix 2 [file jmir_v22i10e17499_app2.png]

Appendix 9: Histograms


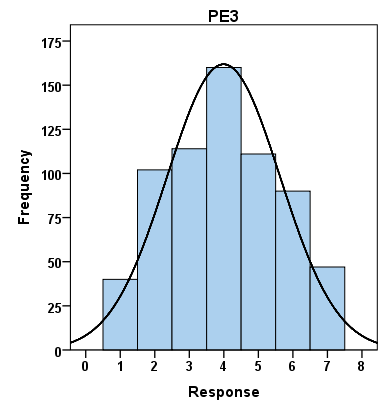

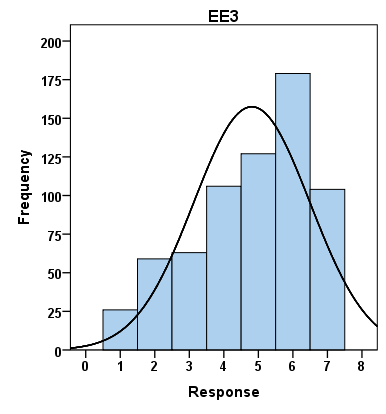

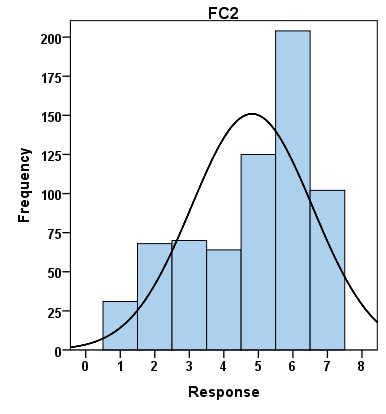

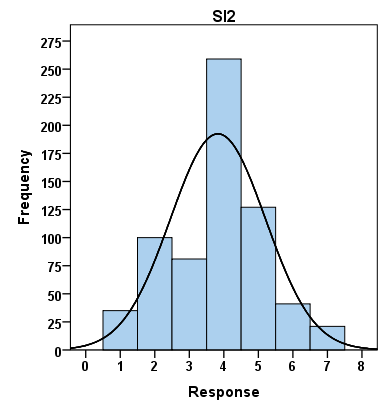

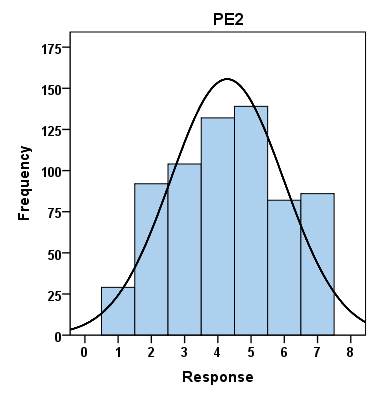

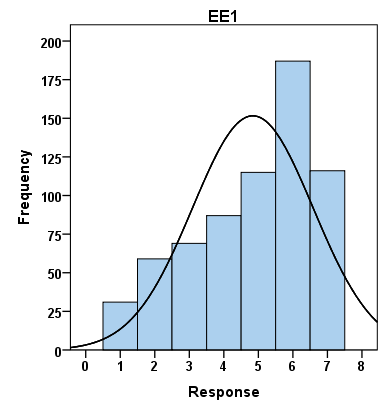

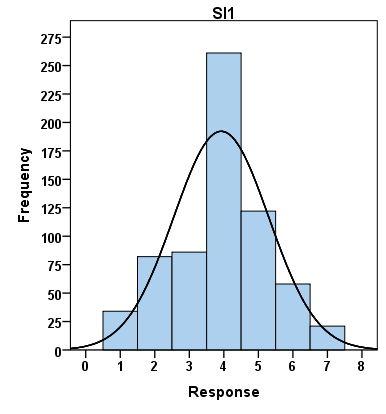

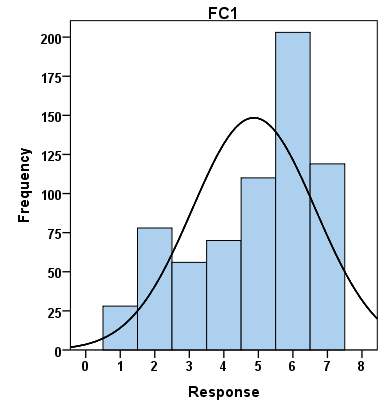

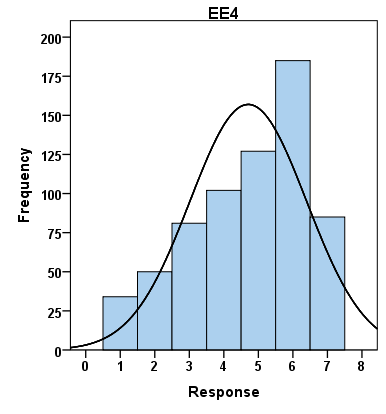

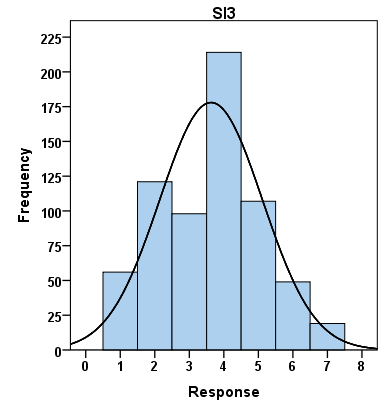

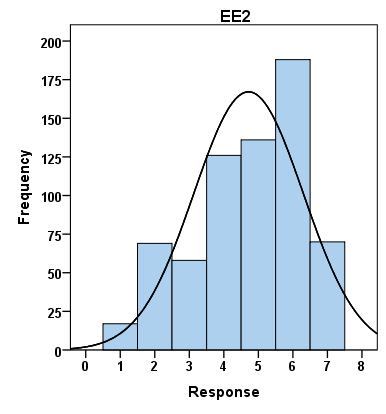

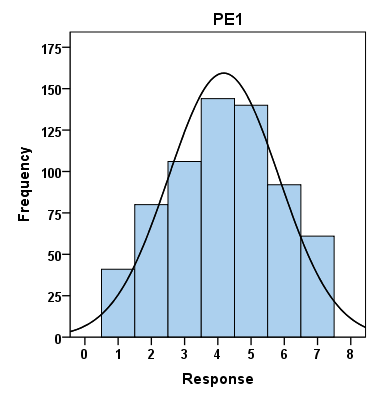

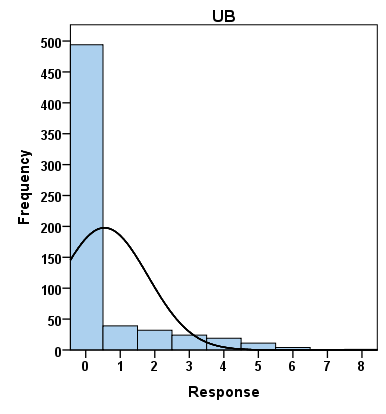

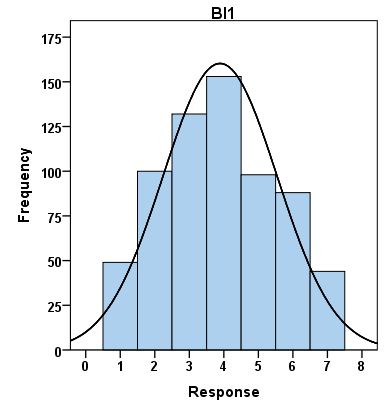

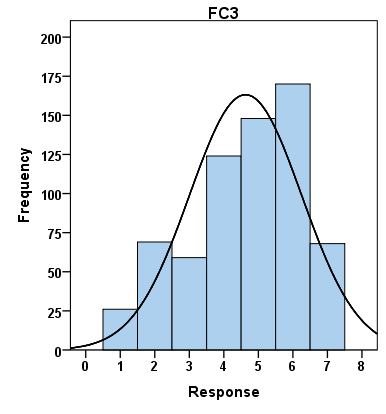

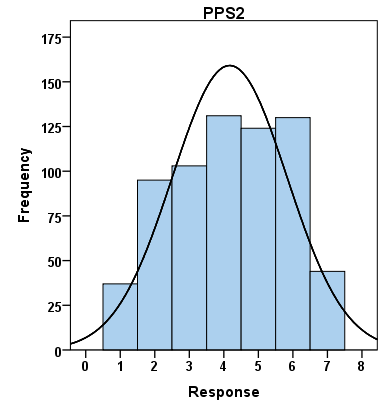

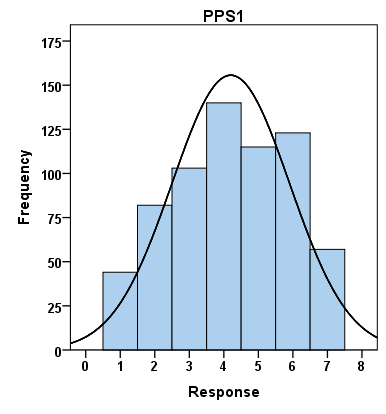

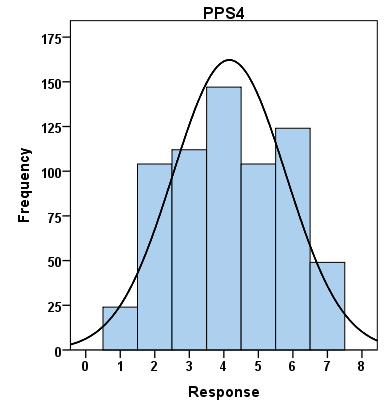

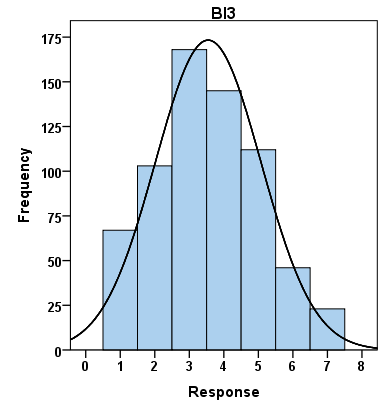

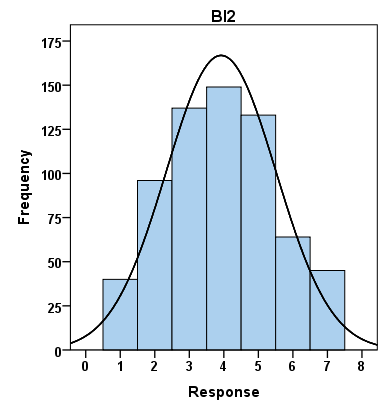

Supplement: Multimedia Appendix 9 [file jmir_v22i10e17499_app9.docx]

Appendix 11: Scatterplot graphs


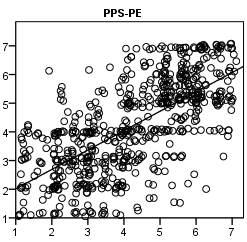

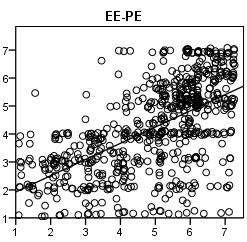

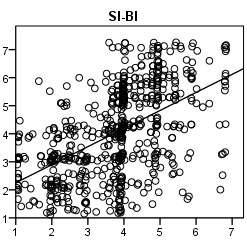

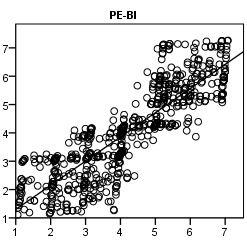

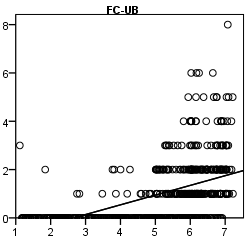

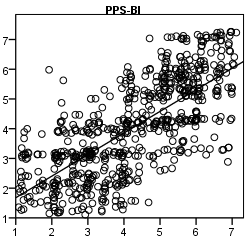

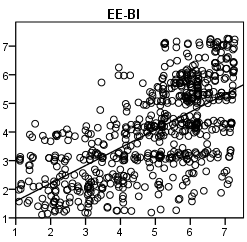

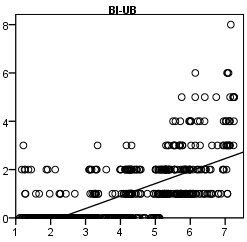

Supplement: Multimedia Appendix 11 [file jmir_v22i10e17499_app11.docx]
