# Supplementary material for: Patients’ Adoption of Electronic Personal Health Records in England: Secondary Data Analysis
Source: J Med Internet Res. 2020 Oct 7;22(10):e17499. doi: 10.2196/17499 (PMC7578819; doi:10.2196/17499)
Supplement: Multimedia Appendix 3 [file jmir_v22i10e17499_app3.docx]

**Appendix 3: Justification of Adaptation of UTAUT**

**Direct effect of perceived privacy and security (PPS)**

In the context of ePHRs, perceived privacy and security may be defined as the extent to which patients perceive that ePHRs are secure and able to keep their information private [1, 2]. Strictly speaking, individuals who have positive perception about the privacy and security of a technology are more likely to intend to adopt it [3, 4]. This relationship has been demonstrated by many studies in the contexts of ePHRs [e.g. 2, 5, 6-8]. This means that patients who believe that Patient Online is secure and maintain their privacy tend more to intend to use it. So, this study theorises that perceived privacy and security positively influences patients’ intention to use Patient Online.

**Mediating effect of perceived privacy and security (PPS)**

The indirect effect of perceived privacy and security on behavioural intention through performance expectancy was not proposed in UTAUT. However, this indirect effect was proposed in the current study as patients’ perceptions about the usefulness of the system are influenced considerably by their perceptions about privacy and security of their data in the system [2, 9-12]. This means that patients who perceive that Patient Online is secure and able to maintain their privacy are more likely to perceive it as a useful system, thereby, they are more likely to intend to use it. Thus, this study posits that performance expectancy positively mediates the positive relationship between perceived privacy and security and behavioural intention.

**Mediating effect of effort expectancy (EE)**

Davis [13] proposed in his well-known model (i.e. TAM) that there is indirect effect of perceived ease of use (i.e. effort expectancy) on behavioural intention through perceived usefulness (i.e. performance expectancy). This indirect effect may be attributed to the fact that individuals’ perceptions about usefulness of the system are influenced considerably by their perceptions about ease of use of that system [2, 14-17]. This indirect effect of effort expectancy was shown in two studies in the context of consumer health information technologies (CHITs) [18, 19]. This means that patients who perceive Patient Online as an easy to use system are more likely to perceive it as a useful system, thereby, they are more likely to intend to use it. Therefore, this study theorises that performance expectancy positively mediates the positive relationship between effort expectancy and behavioural intention.

**Moderating effect of age**

Laric, Pitta [20] argued that older individuals are more likely to concern about privacy of their data. They attributed this argument to the fact that older people are more likely to suffer from illnesses and diseases, thereby, they may be more worried about their privacy than younger people. Moreover, this effect of age on perceived privacy and security may reflect the fact that older people tend more to excessively doubt and not trust new technologies [21-24]. In the context of ePHRs, this relationship was empirically supported by Baird [25] and Richards [17]. In line with these findings, the following hypothesis is postulated: Age positively moderates the positive relationship between perceived privacy & security and behavioural intention, such that the influence is stronger for older patients.

**Moderating effect of sex**

Laric, Pitta [20] argued that females tend more to concern about the privacy of their data then females. This may reflect the fact that females tend more to excessively doubt and not trust technologies than males [22]. In the context of ePHRs, Richards [17] found a significant difference between males and females in their perception of privacy and security of ePHRs. In line with this, the following hypothesis is postulated: sex moderates the positive relationship between perceived privacy & security and behavioural intention, such that the influence is stronger for females.

**Moderating effect of education**

Broadly speaking, individuals with higher level of education are more likely to be healthier and have no functional and mental limitations [26-28]. In addition, healthier people are less likely to perceive that ePHRs are useful for them [2, 26, 29]. Consequently, it can be supposed that education level negatively moderates the effect of performance expectancy on intention to use [26]. In the context of ePHRs, it has been demonstrated that patients with lower level of education perceive usefulness of ePHRs in a different way than those with higher level of education [17, 26, 30]. Accordingly, this study proposes that education level negatively moderates the positive relationship between performance expectancy and behavioural intention, such that the influence is stronger for patients with lower level of education.

It has been demonstrated that effort expectancy is affected by education level [31, 32]. To be more precise, people with higher level of education are less likely to put into consideration the ease of use of a technology before adopting it. This effect of education may be attributed to several facts. Firstly, people with higher level of education generally are more likely to use the internet and have higher level of health literacy [33-38]. Secondly, people with higher level of education usually have less computer anxiety [39-42], thereby, they are less likely to be worried about ease of use of new technology [39, 43-46]. Lastly, people with higher level of education have more ability to learn a new innovation [31]. In the context of ePHRs, this effect of education has been shown by Daglish [30], Noblin [16], and Richards [17]. As a consequence, this study hypothesises that education level negatively moderates the positive relationship between effort expectancy and behavioural intention, such that the influence is stronger for patients with lower level of education.

According to Jian, Syed-Abdul [47], people with high level of education are more likely to concern about their privacy. Reasonably, this may result from the fact that individuals with higher level of education have more awareness about threats of cyber attacks on breaching their privacy. In the context of ePHRs, this effect of education was found empirically by Richards [17]. Thus, this study proposes that education level positively moderates the positive relationship between perceived privacy & security and behavioural intention, such that the influence is stronger for patients with higher level of education.

By and large, people with lower level of education are less likely to use the internet and have lower level of health literacy [33-38]. Furthermore, people with lower level of education usually have more computer anxiety [39-42]. Consequently, it is reasonable to consider that people with lower level of education tend more to place emphasis on availability of sufficient external support when adopting new technologies. This study proposes that education level negatively moderates the positive relationship between facilitating conditions and use behaviour, such that the influence is stronger for patients with lower level of education.

**Moderating effect of income**

It has been shown that income level affects the perceived usefulness of technology [32, 48]. As people with higher income are more likely to be busier, they tend more to concern about the usefulness of technology before using it [48]. Thus, it can be thought that the association between performance expectancy and behavioural intention is stronger among people with higher income. This association was empirically shown in the ePHRs context by Daglish [30] and Richards [17]. Therefore, this study posits that income positively moderates the positive relationship between performance expectancy and behavioural intention, such that the influence is stronger for patients with higher income.

Generally speaking, people with lower income are less likely to be able to afford internet access and latest technologies [36, 37, 48], and this may make those people having higher computer/ technology anxiety [48, 49]. Therefore, it can be inferred that people with low income are more likely to worry about ease of use of technology before using it. This effect of income was empirically demonstrated in the ePHRs context by Daglish [30] and Richards [17]. Thus, this study claims that income negatively moderates the positive relationship between effort expectancy and behavioural intention, such that the influence is stronger for patients with lower income.

It has been shown that people with lower income are more likely to concern about online information privacy [48, 50, 51]. This may result from the fact that individuals with low income are more likely to have health problems and illnesses [26, 28], thereby, they are more likely to concern about the privacy of their health information [20]. Thus, it can be thought that the association between perceived privacy and security and behavioural intention is stronger among people with lower income. In the context of ePHR, this effect of income was demonstrated by Richards [17]. Consequently, this study hypothesises that income negatively moderates the positive relationship between perceived privacy and security and behavioural intention, such that the influence is stronger for patients with lower income.

As indicated previously, people with lower income are less likely to be able to afford internet access and latest technologies [36, 37, 48], and this may make those people having higher computer/ technology anxiety [48, 49]. Therefore, it can be inferred that people with low income are more likely to worry about availability of facilitating conditions before adopting a technology. Hence, this study posits that income negatively moderates the positive relationship between facilitating conditions and use behaviour, such that the influence is stronger for patients with lower income.

**Moderating effect of internet access**

Reasonably, patients who have internet access are more likely to be internet users and, thereby, they are more likely to have less computer anxiety and higher self-efficacy. As mentioned before, individuals with less computer anxiety and higher self-efficacy are less likely to perceive technology easy to use [2, 39, 43-46, 52], thereby, they are less likely to worry about ease of use of technology before using it. Accordingly, this study proposes that internet access moderates the positive relationship between effort expectancy and behavioural intention, such that the influence is stronger for patients without internet access.

As mentioned above, patients without internet access are more likely to high computer anxiety and low computer self-efficacy, thereby, they may tend more to place emphasis on availability of sufficient external support when adopting new technologies. It is can be inferred that the relationship between facilitating conditions and use behaviour is stronger among patients without internet access. Subsequently, this study hypothesises that internet access moderates the positive relationship between facilitating conditions and use behaviour, such that the influence is stronger for patients without internet access.

**References**

1. Gartrell K. Factors associated with electronic personal health record use among registered nurses for their own health management. Maryland, USA: University of Maryland; 2014.

2. Rao M. Factors affecting health care technology use in baby boomers: a quantitative study: Northcentral University; 2014.

3. Featherman MS, Pavlou PA. Predicting e-services adoption: a perceived risk facets perspective. International Journal of Human-Computer Studies. 2003;59(4):451-74.

4. Nicolaou AI, McKnight DH. Perceived information quality in data exchanges: effects on risk, trust, and intention to use. Information Systems Research. 2006;17(4):332-51.

5. Ozok AA, Wu H, Gurses AP. Exploring patients’ use intention of personal health record systems: implications for design. International Journal of Human–Computer Interaction. 2017;33(4):265-79. doi: 10.1080/10447318.2016.1277637.

6. Patel VN, Dhopeshwarkar RV, Edwards A, Barron Y, Likourezos A, Burd L, et al. Low-income, ethnically diverse consumers' perspective on health information exchange and personal health records. Informatics for Health & Social Care. 2011;36(4):233-52. PMID: 919899848; 201201896.

7. Patel VN, Dhopeshwarkar RV, Edwards A, Barron Y, Sparenborg J, Kaushal R. Consumer support for health information exchange and personal health records: a regional health information organization survey. J Med Syst. 2012 Jun;36(3):1043-52. PMID: 20703633. doi: 10.1007/s10916-010-9566-0.

8. Whetstone M, Goldsmith R. Factors influencing intention to use personal health records. International Journal of Pharmaceutical and Healthcare Marketing. 2009;3(1):8-25. doi: 10.1108/17506120910948485.

9. Archer N, Cocosila M. Canadian patient perceptions of electronic personal health records: An empirical investigation. Commun Assoc Info Syst. 2014;34(1):389-406.

10. Emani S, Yamin CK, Peters E, Karson AS, Lipsitz SR, Wald JS, et al. Patient perceptions of a personal health record: a test of the diffusion of innovation model. Journal of medical Internet research. 2012;14(6):1-15. PMID: 23128775. doi: 10.2196/jmir.2278.

11. Feistel G. Technology acceptance model: factors influencing consumers' intent to use electronic personal health records: Central Michigan University; 2014.

12. Mekawie NM. Factors affecting adoption of eHealth in Egypt: Middlesex University; 2013.

13. Davis FD. Perceived usefulness, perceived ease of use, and user acceptance of information technology. Management Information Systems Quarterly. 1989;13(3):319-40.

14. Goff CR. Predictors of patient portal use: Patient engagement through meaningful use: Capella University; 2016.

15. Lazard AJ, Watkins I, Mackert MS, Xie B, Stephens KK, Shalev H. Design simplicity influences patient portal use: the role of aesthetic evaluations for technology acceptance. Journal of the American Medical Informatics Association. 2016;23(e1):e157-e61. doi: 10.1093/jamia/ocv174.

16. Noblin A. Intention to use a personal health record (PHR) a cross sectional view of the characteristics and opinions of patients of one internal medicine practice: University of Central Florida; 2010.

17. Richards R. A study of the intent to fully utilize electronic personal health records in the context of privacy and trust: University of North Texas; 2012.

18. Hsu C, Lee M, Su C. The role of privacy protection in healthcare information systems adoption. J Med Syst. 2013;37(5):1-12.

19. Or CKL, Karsh BT, Severtson DJ, Burke LJ, Brown RL, Brennan PF. Factors affecting home care patients' acceptance of a web-based interactive self-management technology. Journal of the American Medical Informatics Association. 2011 Jan-Feb;18(1):51-9. PMID: 21131605. doi: 10.1136/jamia.2010.007336.

20. Laric MV, Pitta DA, Katsanis LP. Consumer concerns for healthcare information privacy: a comparison of US and Canadian perspectives. Research in Healthcare Financial Management. 2009;12(1):93-111.

21. Castle E, Eisenberger NI, Seeman TE, Moons WG, Boggero IA, Grinblatt MS, et al. Neural and behavioral bases of age differences in perceptions of trust. Proceedings of the National Academy of Sciences. 2012;109(51):20848-52.

22. Faqih KM, Jaradat M. Mobile healthcare adoption among patients in a developing country environment: exploring the influence of age and gender differences. International Business Research. 2015;8(9):142-74.

23. Peter J, Valkenburg PM. Adolescents’ online privacy: toward a developmental perspective. In: Trepte S, Reinecke L, editors. Privacy online. Berlin, Germany: Springer; 2011. p. 221-34.

24. Yao MZ, Rice RE, Wallis K. Predicting user concerns about online privacy. Journal of the Association for Information Science and Technology. 2007;58(5):710-22.

25. Baird A. Extending adoption of innovation theory with consumer influence the case of personal health records (PHRs) and patient portals: Arizona State University; 2012.

26. Beenkens FHC. Acceptance of e-health technology: a patient perspective: Delft University of Technology; 2011.

27. Hoogendijk E, van Groenou MB, van Tilburg T, Deeg D. Educational differences in functional limitations: comparisons of 55–65-year-olds in the Netherlands in 1992 and 2002. International Journal of Public Health. 2008;53(6):281-9.

28. Jacobsen L, Kent M, Lee M, Mather M. America’s aging population. Population Bulletin. 2011;66(1):1-16.

29. Liu LS, Shih PC, Hayes GR, editors. Barriers to the adoption and use of personal health record systems. iConference 2011; 2011; Seattle, USA: ACM Digital library

30. Daglish D. Electronic personal health records: a matter of trust: McMaster University; 2013.

31. Agarwal R, Prasad J. Are individual differences germane to the acceptance of new information technologies? Decision Sciences. 1999;30(2):361-91.

32. Porter CE, Donthu N. Using the technology acceptance model to explain how attitudes determine Internet usage: the role of perceived access barriers and demographics. Journal of Business Research. 2006;59(9):999-1007.

33. Baker L, Wagner TH, Singer S, Bundorf MK. Use of the Internet and e-mail for health care information: results from a national survey. Journal of the American Medical Association. 2003;289(18):2400-6.

34. Liebermann Y, Stashevsky S. Perceived risks as barriers to Internet and e-commerce usage. Qualitative Market Research: An International Journal. 2002;5(4):291-300.

35. Paasche‐Orlow MK, Parker RM, Gazmararian JA, Nielsen‐Bohlman LT, Rudd RR. The prevalence of limited health literacy. Journal of General Internal Medicine. 2005;20(2):175-84.

36. Rainie L. Internet, broadband, and cell phone statistics. Pew Internet & American Life Project; 2010 [22 March 2017]; 1-16]. Available from: <http://www.pewinternet.org/files/>.

37. Rhee KY, Kim W. The adoption and use of the Internet in South Korea. Journal of Computer-Mediated Communication. 2004;9(4):1-8.

38. Scott TL, Gazmararian JA, Williams MV, Baker DW. Health literacy and preventive health care use among Medicare enrollees in a managed care organization. Medical Care. 2002;40(5):395-404.

39. Ellis RD, Allaire JC. Modeling computer interest in older adults: the role of age, education, computer knowledge, and computer anxiety. Human Factors. 1999;41(3):345-55.

40. Gutek BA, Bikson TK. Differential experiences of men and women in computerized offices. Sex Roles. 1985;13(3-4):123-36.

41. Howard GS, Smith RD. Computer anxiety in management: myth or reality? Communications of the ACM. 1986;29(7):611-5.

42. Igbaria M, Parasuraman S. A path analytic study of individual characteristics, computer anxiety and attitudes toward microcomputers. Journal of Management. 1989;15(3):373-88.

43. Igbaria M, Iivari J. The effects of self-efficacy on computer usage. Omega. 1995;23(6):587-605.

44. Lai T, Larson EL, Rockoff ML, Bakken S. User Acceptance of HIV TIDES—tailored interventions for management of depressive symptoms in persons living with HIV/AIDS. J Am Med Informatics Assoc. 2008;15(2):217-26.

45. Torres CA. Examining the role of anxiety and apathy in health consumers' intentions to use patient health portals for personal health information management. United States Florida State University; 2011.

46. Venkatesh V. Determinants of perceived ease of use: integrating control, intrinsic motivation, and emotion into the technology acceptance model. Information Systems Research. 2000;11(4):342-65.

47. Jian W, Syed-Abdul S, Sood SP, Lee P, Hsu M, Ho CH, et al. Factors influencing consumer adoption of USB-based personal health records in taiwan. BMC Health Services Research. 2012;12(1):1-8.

48. Chawla D, Joshi H. The moderating effect of demographic variables on mobile banking adoption: an empirical investigation. Global Business Review. 2018;19(3):90-113.

49. Lee H, Jeong Cho H, Xu W, Fairhurst A. The influence of consumer traits and demographics on intention to use retail self-service checkouts. Marketing Intelligence & Planning. 2010;28(1):46-58.

50. Hernández B, Jiménez J, José Martín M. Age, gender and income: do they really moderate online shopping behaviour? Online Information Review. 2011;35(1):113-33.

51. Zukowski T, Brown I, editors. Examining the influence of demographic factors on internet users' information privacy concerns. 2007 Annual Research Conference of the South African Institute of Computer Scientists and Information Technologists on IT Research in Developing Countries; 2007 2-3 October 2007 Port Elizabeth, South Africa: ACM Digital Library.

52. Venkatesh V, Bala H. Technology acceptance model 3 and a research agenda on interventions. Decision Sciences. 2008;39(2):273-315.
