# Supplementary material for: Patients’ Adoption of Electronic Personal Health Records in England: Secondary Data Analysis
Source: J Med Internet Res. 2020 Oct 7;22(10):e17499. doi: 10.2196/17499 (PMC7578819; doi:10.2196/17499)
Supplement: Multimedia Appendix 4 [file jmir_v22i10e17499_app4.docx]

Appendix 4: Conceptual definitions of constructs

| Constructs | Conceptual definitions |
| --- | --- |
| Performance expectancy | The degree to which patients believe that using Patient Online will enable them to attain several gains |
| Effort expectancy | The degree to which patients perceive that using Patient Online is easy to use |
| Social influence | The degree to which patients perceive that important others believe they should use Patient Online |
| Facilitating conditions | The degree to which patients believe that an organisational and technical infrastructure exists to support the use of Patient Online |
| Perceived privacy and security | The degree to which patients believe that Patient Online is safe from intrusion and personal information is protected |
| Behavioural intention | The degree to which patients plan to use or not use Patient Online |
| Use behaviour | Number of times a patient logged in to Patient Online during six months after completing the questionnaire |
| Age | The period of time (in years) that a patient has lived when completing the questionnaire |
| Sex | The patient’s state of being either male or female |
| Educational level | The highest level of education a patient has achieved or been studying when completing the questionnaire |
| Income | The amount of money that a whole patient’s household earns per year |
| Ethnicity | The patient’s state of belonging to a social group that has a common national, racial, or cultural origin |
| Internet access | The patient’s state of having internet access in his/her home. |
