# Supplementary material for: Patients’ Adoption of Electronic Personal Health Records in England: Secondary Data Analysis
Source: J Med Internet Res. 2020 Oct 7;22(10):e17499. doi: 10.2196/17499 (PMC7578819; doi:10.2196/17499)
Supplement: Multimedia Appendix 8 [file jmir_v22i10e17499_app8.docx]

Appendix 8: Participants' characteristics

| Variable | Groups | Respondents (n=624) | | Target population (n=31742) | | Diff^1^ | P-Value^2^ |
| --- | --- | --- | --- | --- | --- | --- | --- |
|  |  | **n** | **%** | **n** | **%** | **%** |  |
| Age, mean (SD) | - | 44.2 (1.89), years | | - | | - | - |
| Age | 18-24 | 107 | 17.1 | 5850 | 18.4 | 1.3 | 0.213 |
|  | 25-34 | 148 | 23.7 | 7031 | 22.2 | 1.5 |  |
|  | 35-44 | 116 | 18.6 | 5030 | 15.8 | 2.8 |  |
|  | 45-54 | 98 | 15.7 | 4656 | 14.7 | 1.0 |  |
|  | 55-64 | 65 | 10.4 | 3944 | 12.4 | 2.0 |  |
|  | 65-74 | 46 | 7.4 | 2813 | 8.90 | 1.5 |  |
|  | 75+ | 44 | 7.1 | 2418 | 7.60 | 0.5 |  |
| Sex | Male | 293 | 46.9 | 16097 | 50.7 | 3.8 | 0.063 |
|  | Female | 331 | 53.1 | 15645 | 49.3 | 3.8 |  |
| Ethnicity | White | 498 | 79.8 | 25099 | 79.1 | 0.7 | 0.643 |
|  | Asian | 73 | 11.7 | 4203 | 13.2 | 1.5 |  |
|  | Black | 20 | 3.2 | 1013 | 3.2 | 0.0 |  |
|  | Mixed | 26 | 4.1 | 1051 | 3.3 | 0.8 |  |
|  | Others | 7 | 1.2 | 376 | 1.2 | 0.0 |  |
| Income | < 20,000 | 284 | 45.5 | - | - | - | - |
|  | 20,000-29,999 | 80 | 12.8 | - | - | - | - |
|  | 30,000-39,999 | 65 | 10.4 | - | - | - | - |
|  | 40,000-49,999 | 43 | 7 | - | - | - | - |
|  | 50,000-59,999 | 26 | 4.2 | - | - | - |  |
|  | 60,000 or more | 12 | 1.9 | - | - | - | - |
|  | Prefer not to say | 114 | 18.2 | - | - | - |  |
| Education | Up to secondary school | 69 | 11.1 | - | - | - | - |
|  | Secondary school | 147 | 23.6 | - | - | - | - |
|  | College/ Diploma | 165 | 26.4 | - | - | - | - |
|  | Bachelor Degree | 174 | 27.9 | - | - | - | - |
|  | Master Degree | 47 | 7.5 | - | - | - | - |
|  | Doctoral Degree | 22 | 3.5 | - | - | - | - |
| Internet access | Yes | 528 | 84.6 | - | - | - | - |
|  | No | 96 | 15.4 | - | - | - | - |
| *^1^: Absolute difference between respondents and non-respondents (percentage)*  *^2^: Chi-square was used* | | | | | | | |
