# Supplementary material for: Patients’ Adoption of Electronic Personal Health Records in England: Secondary Data Analysis
Source: J Med Internet Res. 2020 Oct 7;22(10):e17499. doi: 10.2196/17499 (PMC7578819; doi:10.2196/17499)
Supplement: Multimedia Appendix 10 [file jmir_v22i10e17499_app10.docx]

Appendix 10: Values of skewness and kurtosis

| Variables | Items | Skewness | Kurtosis |
| --- | --- | --- | --- |
| Performance expectancy | PE1 | -0.10 | -0.79 |
|  | PE2 | -0.04 | -0.92 |
|  | PE3 | 0.05 | -0.82 |
| Effort expectancy | EE1 | -0.60 | -0.68 |
|  | EE2 | -0.52 | -0.61 |
|  | EE3 | -0.57 | -0.61 |
|  | EE4 | -0.55 | -0.63 |
| Social influence | SI1 | -0.13 | -0.15 |
|  | SI2 | -0.09 | -0.19 |
|  | SI3 | 0.05 | -0.56 |
| Facilitating condition | FC1 | -0.65 | -0.75 |
|  | FC2 | -0.64 | -0.71 |
|  | FC3 | -0.50 | -0.61 |
| Perceived privacy and security | PPS1 | -0.14 | -0.91 |
|  | PPS2 | -0.15 | -0.96 |
|  | PPS4 | -0.01 | -0.98 |
| Behavioural intention | BI1 | 0.11 | -0.81 |
|  | BI2 | 0.10 | -0.68 |
|  | BI3 | 0.20 | -0.54 |
| Use Behaviour | UB | 2.59 | 6.53 |
