# Supplementary material for: Patients’ Adoption of Electronic Personal Health Records in England: Secondary Data Analysis
Source: J Med Internet Res. 2020 Oct 7;22(10):e17499. doi: 10.2196/17499 (PMC7578819; doi:10.2196/17499)
Supplement: Multimedia Appendix 12 [file jmir_v22i10e17499_app12.docx]

Appendix 12: Results of Curve Estimation procedure

| Relationship | R Square | F | Sig. | Linearity? |
| --- | --- | --- | --- | --- |
| PE-BI | 0.738 | 1864.213 | .000 | linear |
| EE-BI | 0.409 | 458.916 | .000 | linear |
| SI-BI | 0.274 | 250.135 | .000 | linear |
| PPS-BI | 0.526 | 733.540 | .000 | linear |
| EE-PE | 0.350 | 355.983 | .000 | linear |
| PPS-PE | 0.450 | 541.960 | .000 | linear |
| BI-UB | 0.438 | 515.135 | .000 | linear |
| FC-UB | 0.299 | 282.937 | .000 | linear |
