# Supplementary material for: Patients’ Adoption of Electronic Personal Health Records in England: Secondary Data Analysis
Source: J Med Internet Res. 2020 Oct 7;22(10):e17499. doi: 10.2196/17499 (PMC7578819; doi:10.2196/17499)
Supplement: Multimedia Appendix 13 [file jmir_v22i10e17499_app13.docx]

Appendix 13: Tolerance values

| Dependent variable | Independent variables | Tolerance |
| --- | --- | --- |
| UB | BI | 0.661 |
|  | FC | 0.661 |
| BI | PE | 0.450 |
|  | EE | 0.600 |
|  | SI | 0.658 |
|  | PPS | 0.505 |
| PE | EE | 0.737 |
|  | PPS | 0.737 |
