# Supplementary material for: Patients’ Adoption of Electronic Personal Health Records in England: Secondary Data Analysis
Source: J Med Internet Res. 2020 Oct 7;22(10):e17499. doi: 10.2196/17499 (PMC7578819; doi:10.2196/17499)
Supplement: Multimedia Appendix 14 [file jmir_v22i10e17499_app14.docx]

Appendix 14: Results of Harman’s Single-Factor Test

| **Total Variance Explained** | | | | | | |
| --- | --- | --- | --- | --- | --- | --- |
| Item | Initial Eigenvalues | | | Extraction Sums of Squared Loadings | | |
|  | Total | % of Variance | Cumulative % | Total | % of Variance | Cumulative % |
| 1 | 10.739 | **47.344** | **47.344** | 10.739 | 47.344 | 47.344 |
| 2 | 1.810 | 12.529 | 59.873 | 1.810 | 12.529 | 59.873 |
| 3 | 1.786 | 11.402 | 71.275 | 1.786 | 11.402 | 71.275 |
| 4 | 1.343 | 9.070 | 80.345 | 1.343 | 9.070 | 80.345 |
| **5** | 1.078 | 7.850 | 88.195 | 1.078 | 7.850 | 88.195 |
| 6 | .526 | 2.767 | 90.961 |  |  |  |
| 7 | .233 | 1.228 | 92.190 |  |  |  |
| 8 | .200 | 1.051 | 93.241 |  |  |  |
| 9 | .183 | .961 | 94.202 |  |  |  |
| 10 | .166 | .874 | 95.076 |  |  |  |
| 11 | .156 | .822 | 95.898 |  |  |  |
| 12 | .136 | .717 | 96.616 |  |  |  |
| 13 | .116 | .612 | 97.227 |  |  |  |
| 14 | .107 | .564 | 97.791 |  |  |  |
| 15 | .101 | .533 | 98.324 |  |  |  |
| 16 | .096 | .507 | 98.831 |  |  |  |
| 17 | .088 | .463 | 99.294 |  |  |  |
| 18 | .070 | .371 | 99.664 |  |  |  |
| 19 | .064 | .336 | 100.000 |  |  |  |
