# Supplementary material for: Patients’ Adoption of Electronic Personal Health Records in England: Secondary Data Analysis
Source: J Med Internet Res. 2020 Oct 7;22(10):e17499. doi: 10.2196/17499 (PMC7578819; doi:10.2196/17499)
Supplement: Multimedia Appendix 16 [file jmir_v22i10e17499_app16.docx]

| **Constructs** | **Cronbach’s alpha (α)^a^** | **Composite Reliability (CR)^a^** | **Average Variance Extracted (AVE)^b^** |
| --- | --- | --- | --- |
| **Performance expectancy** | 0.962 | 0.962 | 0.895 |
| **Effort expectancy** | 0.961 | 0.962 | 0.863 |
| **Social influence** | 0.946 | 0.948 | 0.858 |
| **Facilitating conditions** | 0.940 | 0.942 | 0.843 |
| **Perceived privacy and security** | 0.941 | 0.942 | 0.845 |
| **Behavioural intention** | 0.962 | 0.963 | 0.898 |
| *^a^Recommneded value of ≥0.70.*  *^b^Recommneded value ≥0.50.* | | | |

Appendix 16: Results of construct reliability
