# Supplementary material for: Patients’ Adoption of Electronic Personal Health Records in England: Secondary Data Analysis
Source: J Med Internet Res. 2020 Oct 7;22(10):e17499. doi: 10.2196/17499 (PMC7578819; doi:10.2196/17499)
Supplement: Multimedia Appendix 17 [file jmir_v22i10e17499_app17.docx]

| **Latent Constructs** | **Items** | **Factor loading^a^** | **Average Variance Extracted (AVE)^b^** |
| --- | --- | --- | --- |
| **Performance expectancy (PE)** | **PE1** | 0.97 | 0.895 |
|  | **PE2** | 0.95 |  |
|  | **PE3** | 0.92 |  |
| **Effort expectancy (EE)** | **EE1** | 0.95 | 0.863 |
|  | **EE2** | 0.92 |  |
|  | **EE3** | 0.95 |  |
|  | **EE4** | 0.90 |  |
| **Social influences (SI)** | **SI1** | 0.94 | 0.858 |
|  | **SI2** | 0.96 |  |
|  | **SI3** | 0.88 |  |
| **Facilitating conditions (FC)** | **FC1** | 0.97 | 0.843 |
|  | **FC2** | 0.93 |  |
|  | **FC3** | 0.86 |  |
| **Perceived privacy & security (PPS)** | **PPS1** | 0.95 | 0.845 |
|  | **PPS2** | 0.94 |  |
|  | **PPS3** | 0.86 |  |
| **Behavioural intention (BI)** | **BI1** | 0.97 | 0.898 |
|  | **BI2** | 0.95 |  |
|  | **BI3** | 0.91 |  |
| *^a^Recommneded value of ≥0.70.*  *^b^Recommneded value ≥0.50.* | | | |

Appendix 17: Results of convergent validity
