# Supplementary material for: Patients’ Adoption of Electronic Personal Health Records in England: Secondary Data Analysis
Source: J Med Internet Res. 2020 Oct 7;22(10):e17499. doi: 10.2196/17499 (PMC7578819; doi:10.2196/17499)
Supplement: Multimedia Appendix 18 [file jmir_v22i10e17499_app18.docx]

Appendix 18: Inter-correlation coefficients and squared roots of AVE

| **Latent constructs** | **PE^a^** | **EE^b^** | **SI^c^** | **FC^d^** | **PPS^e^** | **BI^f^** |
| --- | --- | --- | --- | --- | --- | --- |
| **PE^a^** | **0.946** |  |  |  |  |  |
| **EE^b^** | 0.454 | **0.929** |  |  |  |  |
| **SI^c^** | 0.647 | 0.501 | **0.926** |  |  |  |
| **FC^d^** | 0.563 | 0.837 | 0.530 | **0.918** |  |  |
| **PPS^e^** | 0.538 | 0.525 | 0.701 | 0.541 | **0.919** |  |
| **BI^f^** | 0.493 | 0.506 | 0.484 | 0.573 | 0.621 | **0.947** |
| *^a^Performace expectancy.*  *^b^Effort expectancy.*  *^c^Social influence.*  *^d^Facilitating conditions.*  *^e^Percevied Privacy and Security.*  *^f^Behavioural intention.* | | | | | | |
