# Supplementary material for: Patients’ Adoption of Electronic Personal Health Records in England: Secondary Data Analysis
Source: J Med Internet Res. 2020 Oct 7;22(10):e17499. doi: 10.2196/17499 (PMC7578819; doi:10.2196/17499)
Supplement: Multimedia Appendix 19 [file jmir_v22i10e17499_app19.docx]

| **Items**  **Constructs** | **PE^a^** | **EE^b^** | **SI^c^** | **FC^d^** | **PPS^e^** | **BI^f^** |
| --- | --- | --- | --- | --- | --- | --- |
| **PE1** | **0.969** | 0.555 | 0.485 | 0.524 | 0.627 | 0.811 |
| **PE2** | **0.953** | 0.545 | 0.477 | 0.515 | 0.616 | 0.797 |
| **PE3** | **0.916** | 0.524 | 0.458 | 0.495 | 0.592 | 0.766 |
| **EE1** | 0.543 | **0.949** | 0.431 | 0.503 | 0.468 | 0.589 |
| **EE2** | 0.526 | **0.918** | 0.417 | 0.486 | 0.453 | 0.570 |
| **EE3** | 0.542 | **0.947** | 0.430 | 0.502 | 0.467 | 0.588 |
| **EE4** | 0.516 | **0.901** | 0.409 | 0.477 | 0.444 | 0.559 |
| **SI1** | 0.469 | 0.426 | **0.938** | 0.493 | 0.454 | 0.474 |
| **SI2** | 0.479 | 0.435 | **0.958** | 0.503 | 0.464 | 0.484 |
| **SI3** | 0.441 | 0.400 | **0.881** | 0.463 | 0.427 | 0.446 |
| **FC1** | 0.522 | 0.511 | 0.507 | **0.965** | 0.519 | 0.543 |
| **FC2** | 0.502 | 0.492 | 0.488 | **0.928** | 0.499 | 0.522 |
| **FC3** | 0.465 | 0.455 | 0.451 | **0.859** | 0.462 | 0.484 |
| **PPS1** | 0.616 | 0.470 | 0.461 | 0.513 | **0.953** | 0.667 |
| **PPS2** | 0.611 | 0.466 | 0.458 | 0.508 | **0.945** | 0.662 |
| **PPS4** | 0.555 | 0.423 | 0.416 | 0.462 | **0.858** | 0.601 |
| **BI1** | 0.814 | 0.604 | 0.492 | 0.548 | 0.682 | **0.973** |
| **BI2** | 0.798 | 0.592 | 0.482 | 0.537 | 0.668 | **0.954** |
| **BI3** | 0.765 | 0.567 | 0.462 | 0.514 | 0.64 | **0.914** |
| *^a^Performace expectancy.*  *^b^Effort expectancy.*  *^c^Social influence.*  *^d^Facilitating conditions.*  *^e^Percevied Privacy and Security.*  *^f^Behavioural intention.* | | | | | | |

Appendix 19: Item loadings and cross-loadings
