# Supplementary material for: Patients’ Adoption of Electronic Personal Health Records in England: Secondary Data Analysis
Source: J Med Internet Res. 2020 Oct 7;22(10):e17499. doi: 10.2196/17499 (PMC7578819; doi:10.2196/17499)
Supplement: Multimedia Appendix 20 [file jmir_v22i10e17499_app20.docx]

| Fit indices | Recommended value | Estimated value |
| --- | --- | --- |
| χ2/*df*^a^ | **1-3** | 1.470 |
| GFI^b^ | **≥ 0.95** | 0.966 |
| AGFI^c^ | **≥ 0.90** | 0.954 |
| RMSEA^d^ | **< 0.05** | 0.027 |
| PCLOSE^e^ | **≥0.05** | 1.000 |
| SRMR^f^ | **≤0.05** | 0.018 |
| NFI^g^ | **≥ 0.95** | 0.986 |
| CFI^h^ | **≥ 0.95** | 0.995 |
| TLI^i^ | **≥ 0.95** | 0.994 |
| *^a^χ2/df: relative chi-square (df)*  *^b^GFI: goodness-of-fit index.*  *^c^AGFI: adjusted goodness-of-fit index.*  *^d^RMSEA: root mean square error of approximation.*  *^e^PCLOSE: p of close fit.*  *^f^SRMR: standardized root mean square residual.*  *^g^NFI: normed fit index.*  *^h^CFI: comparative fit index.*  *^i^TLI: Tucker-Lewis index.* | | |

Appendix 20: Results of fit indices of the structural model
